# Visualising harms in publications of randomised controlled trials: consensus and recommendations

**DOI:** 10.1136/bmj-2021-068983

**Published:** 2022-05-16

**Authors:** Rachel Phillips, Suzie Cro, Graham Wheeler, Simon Bond, Tim P Morris, Siobhan Creanor, Catherine Hewitt, Sharon Love, Andre Lopes, Iryna Schlackow, Carrol Gamble, Graeme MacLennan, Chris Habron, Anthony C Gordon, Nikhil Vergis, Tianjing Li, Riaz Qureshi, Colin C Everett, Jane Holmes, Amanda Kirkham, Clare Peckitt, Sarah Pirrie, Norin Ahmed, Laura Collett, Victoria Cornelius

**Affiliations:** 1 Imperial Clinical Trials Unit, School of Public Health, Imperial College London, London, UK; 2 Pragmatic Clinical Trials Unit, Centre for Evaluation and Methods, Wolfson Institute of Population Health, Queen Mary University of London, London, UK; 3 Cambridge Clinical Trials Unit, Cambridge University Hospitals NHS Foundation Trust, Cambridge, UK; 4 MRC Clinical Trials Unit at University College London, Institute of Clinical Trials and Methodology, London, UK; 5 Exeter Clinical Trials Unit, University of Exeter, Exeter, UK; 6 York Trials Unit, University of York, York, UK; 7 CRUK Cancer Trials Centre, University College London, London, UK; 8 Nuffield Department of Population Health, University of Oxford, Oxford, UK; 9 Liverpool Clinical Trials Centre, University of Liverpool, Liverpool, UK; 10 Centre for Health Care Randomised Trials, University of Aberdeen, Aberdeen, UK; 11 Roche Products, Welwyn Garden City, UK; 12 Division of Anaesthetics, Pain Medicine, and Intensive Care, Department of Surgery and Cancer, Imperial College London and Imperial College Healthcare NHS Trust, London, UK; 13 Imperial College London and Imperial NHS Trust, London, UK; 14 Department of Ophthalmology, School of Medicine, University of Colorado Anschutz Medical Campus, Aurora, CO, USA; 15 Clinical Trials Research Unit, Leeds Institute for Clinical Trials Research, University of Leeds, Leeds, UK; 16 Oxford Clinical Trials Research Unit, Centre for Statistics in Medicine, Nuffield Department of Orthopaedics, Rheumatology, and Musculoskeletal Sciences, University of Oxford, Oxford, UK; 17 Cancer Research UK Clinical Trials Unit, University of Birmingham, Birmingham, UK; 18 Royal Marsden Clinical Trials Unit, Royal Marsden NHS Foundation Trust, London, UK; 19 Comprehensive Clinical Trials Unit, University College London, London, UK; 20 Bristol Trials Centre, University of Bristol, Bristol, UK

## Abstract

**Objective:**

To improve communication of harm in publications of randomised controlled trials via the development of recommendations for visually presenting harm outcomes.

**Design:**

Consensus study.

**Setting:**

15 clinical trials units registered with the UK Clinical Research Collaboration, an academic population health department, Roche Products, and *The* *BMJ*.

**Participants:**

Experts in clinical trials: 20 academic statisticians, one industry statistician, one academic health economist, one data graphics designer, and two clinicians.

**Main outcome:**

**measures** A methodological review of statistical methods identified visualisations along with those recommended by consensus group members. Consensus on visual recommendations was achieved (at least 60% of the available votes) over a series of three meetings with participants. The participants reviewed and critically appraised candidate visualisations against an agreed framework and voted on whether to endorse each visualisation. Scores marginally below this threshold (50-60%) were revisited for further discussions and votes retaken until consensus was reached.

**Results:**

28 visualisations were considered, of which 10 are recommended for researchers to consider in publications of main research findings. The choice of visualisations to present will depend on outcome type (eg, binary, count, time-to-event, or continuous), and the scenario (eg, summarising multiple emerging events or one event of interest). A decision tree is presented to assist trialists in deciding which visualisations to use. Examples are provided of each endorsed visualisation, along with an example interpretation, potential limitations, and signposting to code for implementation across a range of standard statistical software. Clinician feedback was incorporated into the explanatory information provided in the recommendations to aid understanding and interpretation.

**Conclusions:**

Visualisations provide a powerful tool to communicate harms in clinical trials, offering an alternative perspective to the traditional frequency tables. Increasing the use of visualisations for harm outcomes in clinical trial manuscripts and reports will provide clearer presentation of information and enable more informative interpretations. The limitations of each visualisation are discussed and examples of where their use would be inappropriate are given. Although the decision tree aids the choice of visualisation, the statistician and clinical trial team must ultimately decide the most appropriate visualisations for their data and objectives. Trialists should continue to examine crude numbers alongside visualisations to fully understand harm profiles.

## Introduction

Well designed graphics are an effective way of communicating messages to a range of audiences and help to identify patterns in data that might otherwise be missed.^1^ In 1983, Tufte stated, “of all methods for analysing and communicating statistical information, well-designed graphics are usually the simplest and at the same time the most powerful.”^2^ In clinical trials, when analysing emerging harm outcomes (ie, non-prespecified events that are reported during the trial and might be unexpected) for which a lot of complex data are collected, visualisations can help to summarise harm profiles (ie, the summary or burden of the cumulative effect of all harm outcomes) and identify potential adverse (drug) reactions. Adverse drug reactions are defined as harm outcomes where a causal relationship between the intervention and event is “at least a reasonable possibility.”^3^ ^4^ Trials can also prespecify events as harm outcomes of interest to follow up. Prespecified events are individual events that are listed in advance as harm outcomes of interest. These events might be known or suspected to be associated with the intervention or be followed-up for reasons of interest, and visualisations can be beneficial here too. Trial reporting guidelines encourage the use of visualisations for exploring harm outcomes, including the CONSORT (consolidated standards of reporting trials) extension to harms, the 2016 recommendations to improve adverse event reporting from industry representatives and journal editors, a pharmaceutical industry standard from the Safety Planning, Evaluation, and Reporting Team, and guidance from regulators on statistical principles in clinical trials (known as ICH E9).^5^ ^6^ ^7^ ^8^ The term adverse event is used interchangeably in the literature to refer to harm outcomes but is technically defined as “any untoward medical occurrence that may present during treatment with a pharmaceutical product but which does not necessarily have a causal relationship with this treatment.”^3^ Potential visualisations for harm outcomes are in abundance but their use in journal articles is limited.^5^ ^6^ ^9^ ^10^ A systematic review from 2018 found that only 12% of journal articles made use of visual summaries for adverse event data; a finding supported by a 2019 survey of the UK Clinical Research Collaboration clinical trial unit statisticians.^11^ ^12^ However, a 2016 survey of pharmaceutical industry statisticians suggested that in-house practice in this sector might differ.^13^ Evidence suggests that journal articles tend to summarise harm outcomes from randomised controlled trials in simple tables of frequencies and percentages, despite the advantages that visualisations offer.^14^

Advances in computer software have improved trialists’ capability to produce visualisations; however, little guidance exists on what and how to visually display complex harm data in journal articles. This has resulted in independent calls from the statistical community for direction on “how to decide which of many possible graphics to draw.”^12^ ^15^ Therefore, with a range of visualisation options available and the increasing ease with which they can be implemented, we sought a consensus to support researchers in their choice of visualisations for randomised controlled trial publications. In collaboration with the UK Clinical Research Collaboration clinical trial unit Statistics Operations Group, we provide recommendations on which visualisations researchers should consider using in the publication of their main research findings.

## Methods

We held a series of consensus meetings with 20 statisticians from 15 UK Clinical Research Collaboration registered clinical trial units, one health economist based at an academic population health department, one industry statistician, and one data graphics designer who is part of the multimedia team at *The BMJ*. All these participants are experienced clinical trialists or have an interest in the visual representation of data, or fit into both categories. Against an agreed framework, the group reviewed and critically evaluated 28 plots proposed for visualising data for harm outcomes and refined these plots as necessary, predominantly focusing on clinical trials of an investigational medicinal product. Examples of each of the candidate plots was produced by use of data from one of four completed parallel arm randomised controlled trials and a synthetic dataset (see supplement 1 for further details). The group sought consensus on the plots to endorse and then developed recommendations. To support researchers analysing and interpreting harm outcomes, we present a decision tree to aid their choice of visualisations. We focused on static plots that allow a comparison between treatment groups, in line with the aims of randomised controlled trials that make such inferences. Supplement 1 provides details of the methods used for identification of the considered plots, the consensus process, and how the recommendations were developed. In this paper, we describe each of the endorsed plots, give an example interpretation, and provide our recommendation. 

### Patient and public involvement

This work forms part of a wider research project that was developed with input from a range of patient representatives. No patient representatives were directly involved in this work, but representatives with experience as clinical trial participants and patient and public involvement advisors reviewed the original proposal and patient and public involvement strategy. We did not speak to patients directly for this research because our focus was to identify the best plots to present in scientific journals with a predominantly scientific readership. The next step is to ask patients for feedback. 

## Results

We provide the endorsed visualisations that researchers should consider using in the publication of their main research findings according to outcome type and number of events (either single outcomes or multiple outcomes simultaneously; fig 1 and for full size images see supplement 2 figs A1-10).

**Fig 1:**
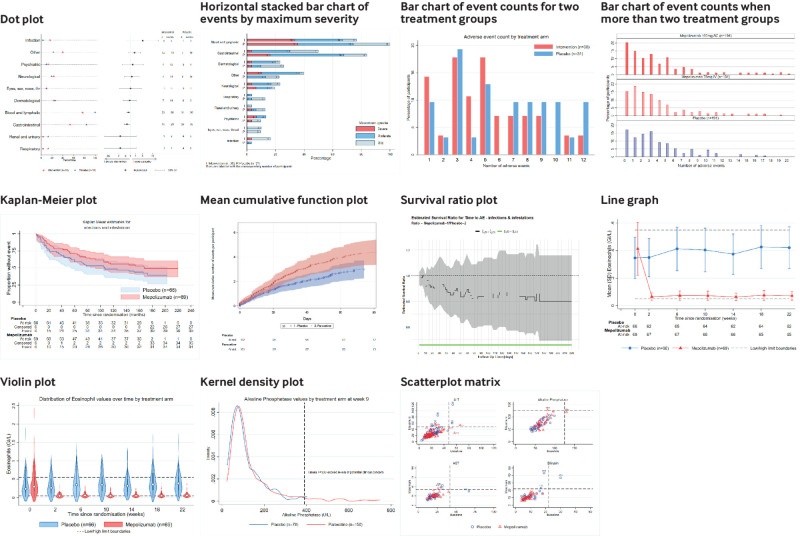
Endorsed visualisations

**Fig 2:**
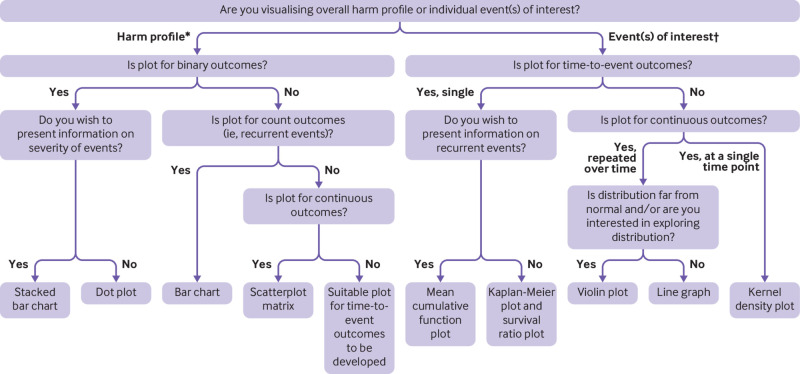
Decision tree to support selection of plots to visualise data for harm outcomes. *Summary of all harm outcomes collected. Individual events include individual emerging events (including adverse events and laboratory or vital sign data indicative of harm) and prespecified events of interest. †Can include a single adverse event (eg, headache), a single category of events that have been grouped together (eg, neurological body system), or an aggregated summary (eg, number of serious adverse events)

Outcome type includes binary harm outcomes, which includes events such as occurrence of a headache or experiencing nausea, count outcomes (ie, the number of occurrences of an event, such as number of headaches experienced over follow-up), time-to-event outcomes (eg, time from treatment to headache), and continuous outcomes (eg, individual results from a blood count). We present endorsed visualisations, according to whether the entire harm profile is assessed or a direct message conveyed about a particular event or events of interest, alongside recommendations for use (table 1). To help trialists decide on which visualisation to use, a decision tree (fig 2) and a summary table of required outcome characteristics (table 2) are provided. Researchers should use these tools when specifying their statistical analysis plan to decide which visualisation they will use, for both prespecified and emerging harm outcomes. Eighteen visualisations were considered but not endorsed (see supplement 3 figs A11-28 for descriptions and potential adaptations discussed).

**Table 1:** Endorsed plots and recommendations for use

Outcome type	Plot	Recommendation
**Visualisations for summarising entire harm profile (viewing differing multiple adverse events)**		
Binary	Dot plot	Use to present a comprehensive summary of the occurrence of multiple binary events
Binary	Stacked bar chart	Use to present information on the occurrence and severity of multiple binary events
Count	Bar chart	Use to present information on event counts
Continuous	Scatterplot matrix	Use in an exploratory setting to help identify any outliers or patterns of interest across multiple continuous outcomes
Time to event	To be developed	No plot endorsed
Visualisations to summarise an event of interest* (viewing a single adverse event)		
Time to event	Kaplan-Meier plot with extended at-risk tables	Use to present information for specific events of interest and to detect either a large between treatment group difference or potential disproportionality over time
Time to event	Survival ratio plot	Use as a signal detection tool to spot departures from unity to help detect potential signals for adverse drug reactions, and alongside the Kaplan-Meier plot to incorporate a direct estimate of between group difference for time-to-event outcomes
Time to event	Mean cumulative function plot	Use to display time-to-event information for recurrent events. Provides a visual summary of the time to expect a certain number of an event to be experienced per participant by treatment group
Continuous	Line graph	Use to describe continuous harm outcomes of interest over time, using an appropriate summary statistic including an indication of variability
Continuous	Violin plot	Use as an alternative plot to the line graph to present a description of continuous harm outcomes of interest over time if, for example, the outcome of interest is far from a normal distribution and/or there is interest in exploring the distribution
Continuous	Kernel density plot	Use to explore and compare an outcome of interest at a specific time point or to investigate how an outcome of interest changes from baseline to either a specific point in time or maximum change over the entire trial period

* Where an event can be a single adverse event (eg, headache) or a single category of events that have been grouped together (eg, neurological body system) or an aggregated summary (eg, number of serious adverse events).

**Table 2:** Summary of characteristics to guide researchers in their choice of plot to visualise data for harm outcomes

Characteristics of outcome to be displayed	Plot
**Binary**	
Multiple outcomes	Dot plot
Multiple outcomes with severity ratings	Stacked bar chart
Count (recurrent) outcome	Bar chart
**Continuous**	
Multiple outcomes	Scatterplot matrix
Single outcome, repeated over time	Line graph
Single outcome, repeated over time with non-normal distribution or interest in exploring the distribution	Violin plot
Single outcome, at single time point	Kernel density plot
**Time-to-event**	
Multiple outcomes	No suitable plot
Single outcome	Kaplan-Meier plot and survival ratio plot
Single, recurrent outcome	Mean cumulative function plot

## Recommendations for multiple binary outcomes

### Dot plot

#### Plot description

The dot plot summarises both the absolute and the relative risk for multiple events (fig 1, supplement 2 fig A1). The left panel displays the percentage of participants who had an event (labelled on the vertical axis) in each treatment group. The central panel displays a measure of comparison—in our example, the relative risk of observing each event in the treatment group compared with the control group is shown, along with corresponding 95% confidence intervals on the log10 scale and a line to show the value of no difference (for relative risks, this is 1). Events on the vertical axis are ordered with the highest risk at the top and decreasing in relative risk at the bottom of the graph. The 95% confidence interval shows the uncertainty around the comparative estimate, and its proximity relative to the value of no difference indicates the strength of evidence against the null hypothesis of no difference in event risk between treatment and control groups. The right panel displays a data table containing the number of participants with at least one event and the number of events by treatment group.

#### Implementation and interpretation

In our example (fig 1, supplement 2 fig A1), the overall impression is that point estimates for the relative summary statistic are evenly distributed on either side of the vertical line but with great differences in levels of precision, shown by the length of the confidence interval, due to the marked differences in the frequencies of the outcome. The largest relative risk communicates increased risk of infection in the intervention group, but the absolute risk and frequencies in the data table show small numbers of participants who had this event. The data show a reduced risk of respiratory events and renal and urinary events in the intervention group; again, the absolute risks and the raw numbers in the data table show only small numbers who had these events. Of note are the estimates for blood and lymphatic disorders and gastrointestinal events, where the relative risks indicate a reduced risk in the intervention group with confidence intervals that do not cross 1. Although these estimates look small compared with the other relative risks, the left side of the plot clearly shows a noticeable difference in absolute numbers, and the data table shows the large numbers of patients who had these events. Therefore, this finding suggests a potential beneficial effect of the intervention on these harm outcomes that might warrant closer inspection.

#### Recommendation

The consensus group unanimously endorsed the dot plot for presenting data for multiple binary outcomes. The dot plot provides a comprehensive presentation of the data that incorporates the traditional table of events. The dot plot was the only visualisation to receive 100% endorsement (see supplement 6 for the endorsement consensus for the other recommended plots).

#### Potential amendments

The relative risk, risk difference, odds ratio, or incident rate ratios (adjusted or unadjusted as desired) can be plotted as the measure of comparison in the central panel of this plot. Some researchers might also prefer to present the data table in the central panel so that it appears alongside the absolute summary. This plot can be presented in grayscale without any loss of meaning. A small number of additional arms can be added for multiarm studies through incorporation of multiple non-overlapping estimates on the same plot (eg, by use of jittering); however, an increase in the number of active treatment groups can lead to incomprehensive distinction between arms.

#### Limitations

Confidence intervals around the relative differences are useful to identify potential signals (that is, information that raises the possibility of a causal relationship between the intervention and event) of harm for further investigation. However, confidence intervals should not be used as a proxy for hypothesis testing, which will increase the chance of finding spurious significant differences resulting from multiple hypothesis tests.^16^ Clinician feedback indicated that trialists should consider varying the horizontal axis range for the absolute summary and scale for the relative summary to ensure clarity without exaggerating effects—for example, presentation of the entire 0-100 scale for the absolute summary might not be appropriate for rare events. When presenting the odds ratios or risk ratios, if no events were reported in one of the treatment groups, a common, simple correction is to add half an event to each group (numerator and denominator). This continuity correction is commonly used but has been shown to be inferior when undertaking meta-analyses for rare events; therefore, alternative corrections might warrant consideration.^17^ ^18^ Although this plot gives a comprehensive overview, some potentially important pieces of information are not included, such as the relative severity of different harm outcomes, and even though recurrent events can be presented using the incident rate ratio, this information cannot be easily displayed on the left panel. In scenarios where information on severity is important, the stacked bar chart can be used, and for recurrent events, the mean cumulative function plot can be used (see later).

#### Software

The dot plot can be produced in Stata by use of the aedot or aedots command, in R with the code available in supplement 4, and in SAS with the code available from the CTSpedia Wiki page (https://www.ctspedia.org/do/view/CTSpedia/ClinAEGraph000). The SAS example does not include code to incorporate the data table.^19^

### Stacked bar chart

#### Plot description

The horizontal stacked bar chart presents the percentage of participants with an event by treatment group and by maximum severity—that is, if a participant had the same event twice, once classified as mild and once as moderate, this participant would be counted once as having a moderate event (fig 1, supplement 2 fig A2). The bars are labelled with the corresponding number of participants. Bars are split by colour gradient to indicate different severity grades, and the total bar height shows the proportion of participants who have had that event at least once. The most severe grade is displayed closest to the vertical axis to allow ease of informal comparison across treatment groups for the most harmful or burdensome events.

#### Implementation and interpretation

In our example (fig 1, supplement 2 fig A2), the most frequent events reported were at least one event of the blood and lymphatic system or gastrointestinal disorders. Although more blood and lymphatic events were noted in the placebo group, the stacked bar chart shows that the proportion in the most severe categories (severe plus moderate) were similar across treatment groups, and the difference in numbers between treatment groups was because of the difference in participants who had a mild event. For gastrointestinal disorders, the stacked bar chart showed that fewer events were recorded for the intervention group across each of the severity grades compared with those in the placebo group. The plot also displays events classified as other that were dominated by severe and moderate events in the intervention group compared with the placebo group, which could warrant closer inspection of the type of events. The stacked bar chart highlights the most frequent events because of the increased physical space that these events occupy. This display contrasts with the dot plot, in which the most frequent events take up the least space in the central panel because of the increased precision and hence narrower confidence intervals around the treatment effect estimate.

#### Recommendations

The stacked bar chart is easy to understand and is useful when it is important to present information on severity of multiple events. This display can be used to informally compare severe or severe plus moderate events or the overall number of events between groups. Treatment groups are recommended to be displayed directly adjacent to each other for each event and horizontally aligned to allow labelling that is easy to read.

#### Potential amendments

This plot can be adapted to multiarm studies, and graduation from black to white is possible without loss of meaning to avoid use of other colours. The single event setting can make use of this graph by replacing events on the vertical axis with representation of time—for example, visits or treatment cycle, an example of which can be found in Thanarajasingam et al.^20^

#### Limitations

Direct comparisons of stacked bars within severity ratings between treatment groups are not possible beyond the segment closest to the vertical axis; however, cumulative comparisons such as severe plus moderate are possible and are perhaps more meaningful. Stacked bar charts promote presentation of information on participants with at least one event at maximum severity rather than number of events, and additional information on repeated events should also be presented. In addition, the effect sizes for differences between groups are not explicitly displayed.

#### Software

Stacked bar charts are easily implemented as standard plots across the variety of statistical packages (graph hbar, Stata; barplot or the ggplot2 package with geom_bar, R; proc gchart, SAS).

## Recommendations for single binary outcomes

### Bar chart

#### Plot description

A bar chart presents information on the number or count of adverse events reported per participant (fig 1, supplement 2 figs A3a and b). Each bar represents the percentage of participants by number of events experienced for each treatment group.

#### Implementation and interpretation

Figure 1 (supplement 2 fig A3a) displays the distribution of multiple events, with higher numbers of multiple events recorded more often for participants in the placebo group than participants in the intervention group. In the alternative figure (fig 1 supplement 2 fig A3b) the distributions indicate that participants in either of the intervention groups had multiple events more often than those in the placebo group.

#### Recommendations

The bar chart is recommended to present information on the number of events experienced. This plot is simple and can be useful to illustrate differences in counts of binary events between treatment groups and is potentially useful to highlight differences in the burden of harm experienced by participants. A bar chart can depict an overall summary of events, such as the total number of serious adverse events, a limited number of events of interest, or a single event of interest. This plot can also be used in an exploratory setting to show the distribution of repeated events.^21^ ^22^ Vertical bars with treatment groups presented alongside each other are the recommended format (fig 1 supplement 2 fig 3a) when comparing two treatment groups. For more than two treatment groups, the recommended alternative is to use separate plots stacked above each other for each group (fig 1 supplement 2 fig 3b).

#### Potential amendments

This plot can be easily adapted to multiarm studies and can be produced in grayscale if necessary. Additionally, bars could be labelled with number of participants to ensure accurate communication.

#### Limitations

Although this plot is helpful for summarising and comparing the overall burden of different treatments, it does not make a distinction between the types of events. Therefore, trialists should still explore and report the individual event data, giving careful consideration as to whether such a plot for overall events could be misleading. In addition, although bar charts could potentially reveal patterns in the data, clinician feedback indicated that subtle differences would be less obvious, and careful consideration of when to use this plot and the accompanying message it supports is needed.

#### Software

Bar charts are easily implemented as standard plots across the variety of statistical packages (graph bar, Stata; barplot or the ggplot2 package with geom_bar, R; proc gchart, SAS).

## Recommendations for single time-to-event outcomes

### Kaplan-Meier plot

#### Plot description

The Kaplan-Meier plot for single time-to-event outcomes shows the cumulative proportion of participants remaining event-free over time by treatment group (fig 1, supplement 2 fig A4). The 95% confidence interval bands indicate the precision of the within group estimates of being event-free. The table below the plot shows the number of participants who remain at risk for the specific event of interest, the cumulative number who have been censored, and the cumulative number who had the event of interest at each discrete time point.

#### Implementation and interpretation

In our example the extended risk table (fig 1) indicates that by the end of follow-up, little difference was noted between treatment groups in the number of participants who had an infection or infestation. However, the event curves show that 50% of the placebo group had this event within about 100 days of randomisation, whereas it took until 160 days after randomisation for 50% of the mepolizumab group to experience the event.

#### Recommendations

We recommend the Kaplan-Meier plot with within group confidence intervals and extended risk table for specific events of interest to detect either a large between treatment group difference or a potential disproportionality over time, especially as adverse drug reactions are often time dependent.

#### Potential amendments

For rare events, trialists might want to reverse the vertical axis to display the cumulative proportion with the event to aid interpretation. This plot can be created in grayscale, with different line styles used to differentiate between groups. Extensions to multiple events or multiarm studies are potentially feasible but can become incomprehensible when displaying multiple overlying confidence bands. Therefore, trialists should consider only plotting the survival estimates with extended risk tables, or present separate plots for comparison of each intervention group, with a common comparator or separate plots for different events.

#### Limitations

Kaplan-Meier plots depict only time-to-first event, failing to consider recurrent events. For clarity in presentation, these graphs are also typically limited to one type of event at a time. To present information on recurrent events over time, a plot of the mean cumulative function (see later) is recommended. Some generic limitations of using time-to-event plots in this setting are discussed later*.*

#### Software

Kaplan-Meier plots are easily implemented as standard plots across a variety of statistical packages. To incorporate the extended risk tables, trialists can use the R package KMunicate and a program for implementation in Stata (https://github.com/sarwarislam/kmunicate_stata).^23^

### Mean cumulative function plot

#### Plot description

For recurrent events or a summary of the total burden of events, the mean cumulative function plot is recommended. This plot is a non-parametric estimate of the mean cumulative number of events per participant (displayed on the vertical axis) as a function of time (horizontal axis) by treatment group (fig 1, supplement 2 fig A5). The 95% confidence interval bands show the precision of the within group estimate. The risk table includes information on the number of participants who remain at risk of an event at discrete time points.

#### Implementation and interpretation

Over the first week after randomisation, the mean number of events per participant is similar across treatment groups, but by day 20 a divergence becomes apparent (fig 1). In the paroxetine group, a mean of two events per participant was observed by day 20, but in the placebo group at that time a mean of approximately 1.5 events per participant was observed. The plot of the mean cumulative function shows the participant burden of recurrent events, highlighting in this example that over follow-up, participants in the paroxetine group had on average a greater number of events than participants in the placebo group, suggesting that some events are associated with the intervention.

#### Recommendations

Unlike the Kaplan-Meier plot, this plot can display information on recurrent events, providing a visual summary of the expected time until a certain number of an event will be recorded per participant by group. This visualisation can show the burden of any event as in the example that we present, or the recurrence of events of special interest. As highlighted in the clinician feedback, these plots are potentially useful when investigating long term treatments for chronic conditions and can provide valuable insight into periods when the treatment might be considered safe or well tolerated. When used to present data for any event, this plot serves as an alternative to the bar chart of counts that incorporates time. This graph also usefully summarises overall burden in place of, or in addition to, summaries of time to discontinuation that are often reported as a proxy for harm.

#### Potential amendments

As with the Kaplan-Meier plot, this plot can be created in grayscale without loss of meaning. Extension to multiarm studies or multiple events is potentially feasible, but displaying multiple overlying confidence bands could make the plot incomprehensible. Similar to the recommendation for the Kaplan-Meier plot, trialists should therefore consider plotting only the mean cumulative function (without confidence bands) and risk table, or present separate plots for comparison of each intervention group with a common comparator, or separate plots for different events.

#### Limitations

For clarity in presentation, mean cumulative function plots are typically limited to one type of event at a time. More generic limitations and cautions of use of time-to-event plots in the harm setting are provided later in this paper*.*

#### Software

The mean cumulative function with confidence interval bands can be implemented using the SAS proc reliability procedure and mcfplot command.

#### Limitations applicable to time-to-event methods

The measure of uncertainty (confidence interval bands) in the Kaplan-Meier plot and the plot of the mean cumulative function are within treatment groups and not between treatment groups, which is the inference of interest in comparative clinical trials. To incorporate an estimate of the between group difference with a measure of uncertainty, the survival ratio plot can be used (see later). Additionally, when time-to-event methods for harm data are used, trialists must remain aware of the limitations around competing risks and consider these when performing the underlying time-to-event analyses. More information on alternative strategies to account for competing risks can be found in Proctor and Schumacher^24^ and include use of appropriate estimates (eg, Aalen-Johnson estimator or Fine and Gray method) to plot the cumulative incident function.

### Survival ratio plot

#### Plot description

The survival ratio plot displays the ratio of non-parametric estimates of the survival probabilities (ie, the probabilities for being event-free in the harm setting) between treatment groups over time along with 95% confidence intervals. Unlike the Kaplan**-**Meier and mean cumulative function plots, this plot allows a direct comparison between treatment groups (fig 1, supplement 2 fig A6). As the plot displays the ratio of survival probabilities over time, departures from unity (point of unity is the survival ratio of 1) indicate potential differences between treatment groups. The green horizontal bar at the bottom of the plot changes to red if the confidence interval excludes unity.^25^

#### Implementation and interpretation

The survival ratio plot (fig 1) depicts a point estimate indicating a greater risk of infection and infestation in the placebo group compared with the intervention group, with a value between 0.9 and 1.0 until day 40 and dropping below 0.9 thereafter. Compared with the Kaplan-Meier plot, this plot shows the confidence band for the between group comparison (rather than within-group confidence intervals in the Kaplan-Meier plot). The confidence band includes the point of unity across all time periods and therefore would not provide sufficient evidence to raise a signal for this event to undergo further investigation.

#### Recommendation

The survival ratio plot would be suitable for signal detection analysis for emerging events because it provides a between group comparison that can be used to spot departures from unity and helps to identify the time that such divergences occur, which can help detect potential signals for adverse drug reactions. For events of specific interest when the focus is on accurately estimating survival probabilities over time, this plot is less suitable. This plot can be presented alongside the Kaplan-Meier plot to show both a relative and an absolute measure.

#### Potential amendments

Our example displays the ratio of survival probabilities estimated using the Kaplan-Meier method; alternatively, the display could show the difference in survival probabilities. As with both the Kaplan-Meier and the mean cumulative function plots, multiple lines can be added to one graph to display estimates for different events or multiple treatment comparisons. 

#### Limitations

As with Kaplan-Meier plots, the survival ratio plot allows for only time-to-first event; therefore, this graph is not suitable for recurrent events. The plot is also limited to one type of event; however, in some situations multiple estimates can be added to the same plot but with the same considerations as plotting multiple lines on the Kaplan-Meier plot. As with other time-to-event plots, competing risks are important to consider when performing the underlying time-to-event analysis, further details of which are discussed above. The confidence interval band of values around the relative differences are useful to detect signals of potential harm for further monitoring, but we are not encouraging hypothesis testing in this setting.^16^ Despite survival ratio plots first being proposed in 2006, little evidence exists of their application in the clinical trial literature; use of this plot will need to be accompanied by a detailed explanation until audiences become more familiar with it and its interpretation.^25^ This postulation was supported in discussions with clinicians, who initially struggled to interpret this plot but who indicated a strong endorsement after further explanation was provided.

#### Software

The survival ratio plot can be implemented in R using the survRatio package with the drsurv function to take the time, censoring indicator, and treatment indicator as inputs. This package returns Kaplan-Meier survival estimates and corresponding confidence intervals to create an object of the survival ratio, survival difference, and pointwise (bootstrap) confidence bands. The ggsurv function is then used to create the plot of the survival ratio and confidence interval bands.

## Recommendations for single continuous outcomes

### Line graph

#### Plot description

In the line graph plot, markers display mean values and vertical lines indicate the standard deviation (not standard error) of raw values at each discrete time point, connected with a line to the point closest in time for each treatment group (fig 1, supplement 2 fig A7). Horizontal reference lines are included to indicate the upper and lower limits of normal values for the outcome, and a table of numbers of participants at risk at each discrete time point is included.

#### Implementation and interpretation

An immediate decrease can be seen in the mean eosinophil count after randomisation in the mepolizumab group (fig 1), and this decreased level is maintained across follow-up. The mean values for the placebo group fluctuate around the baseline value and the error bars exceed the upper limit of normal during follow-up.

#### Recommendations

This plot can be used to describe continuous harm outcomes of interest over time by use of an appropriate summary statistic, together with an indication of variability. This plot can be helpful to identify shifts in distributions between treatment groups and highlight any potential trends; as a result, this display might be better suited to depict clinical outcomes (such as vital signs) rather than blood markers, where clinicians are more often interested in the tails of the distribution (ie, the ends or extremes of the distribution of observed values).

#### Potential adaptations

The summary statistic displayed in this plot should be chosen to reflect each individual dataset and the purpose of the plot, for example, when interest is in presenting descriptions of the distributions, either means and standard deviations or medians and interquartile ranges can be plotted, and if interest is in drawing inferences of between group comparisons, then estimates from mixed effects models for repeated measures with 95% confidence intervals can be presented. This plot can easily incorporate multiple groups or outcomes and can be modified to exclude the use of colour.

#### Limitations

Changes in the tails of the distributions are usually of most interest when monitoring blood markers for harm, and such changes might be difficult to see using this plot. This graph is also unsuitable for skewed distributions; alternative plots for such data are presented below. Appropriate colours and line styles should be considered for clarity, particularly when adapting line graphs to multiarm trials.

#### Software

Line graphs are easily implemented as standard plots across the variety of statistical packages (eg, twoway connected and twoway rspike, Stata; plot and lines or using the ggplot2 package with geom_line and geom_errorbar, R; and proc gplot, SAS).

### Violin plot

#### Plot description

The hollow circle marker on the violin plot indicates the median value, the narrow rectangular boxes indicate the interquartile range, and lines extend from the box to the minimum and maximum points for each group at each time point. These parts are overlaid with kernel density plots (see later), which summarise the distribution of the raw values (fig 1, supplement 2 fig A8). The two horizontal dashed lines indicate the upper and lower limits of normal values. 

#### Implementation and interpretation

At time 0 (randomisation) the distributions were similar across treatment groups, but from week 2 onwards the distribution of values in the mepolizumab group was narrower than in the placebo group (fig 1). The distribution of the values in the placebo group was largely unchanged over time and indicated that a proportion of the participants remained in the upper tail exceeding the upper boundary of normal throughout follow-up. This display indicates a benefit for the mepolizumab group by reducing eosinophil concentrations to within the normal limits.

#### Recommendation

This plot is an alternative to the line graph to describe continuous data that can be used even if the outcome of interest is not normally distributed. Outlying values are displayed and these can be labelled to highlight participants who persistently record values of concern.

#### Possible adaptations

In the current format, information is duplicated because the kernel density plot is mirrored. Presenting only one kernel density would improve clarity and produce a more space efficient plot.

#### Limitations

The violin plot only allows for informal between group comparisons of distributions and does not allow for presentation of formal between group inferences such as the estimates from mixed effects models, which can be presented in a line graph. Adaptations to multiarm trials are not as space efficient as for the line graph. Kernel density estimates for some data might extend to values outside the plausible range—for example, some kernel densities are estimated to be below 0 for eosinophil counts, which is not feasible clinically.

#### Software

The violin plot can be implemented in Stata by use of vioplot or by use of the ggplot2 package in R with geom_violin or SAS proc sgpanel.

### Kernel density plot

#### Plot description

The kernel density plot displays the distribution of a continuous outcome. Data can be for a single time point or a derived change score—for example, the difference between the baseline value and maximum value while receiving treatment (fig 1, supplement 2 fig A9). Vertical reference lines can be included to indicate the upper and lower limits of normal values for the outcome.

#### Implementation and interpretation

Although figure 1 shows that values are similarly distributed in the placebo and paroxetine groups when within the normal range (ie, <390 U/L (6.51 μkat/L)), the plot clearly shows a high alkaline phosphate value for some participants in the paroxetine group through the long right tail. This plot highlights the increased alkaline phosphatase concentrations in some participants taking paroxetine as an important event for closer monitoring in future trials or in the postmarketing setting.

#### Recommendations

The kernel density plot is recommended to explore an outcome of interest at a specific time point or a change score—for example, the change from baseline to a specific point in time or maximum change over the entire trial. This plot can be used to informally compare whole distributions of data between treatment groups and can highlight important differences in these distributions.

#### Potential adaptations

This plot can easily incorporate multiple groups and can be modified to not require use of colour.

#### Limitations

The kernel density plot only allows for informal between group comparisons of distributions and the information on repeated measures is lost, only displaying information for one time point.

#### Software

The kernel density plot can be implemented in Stata by use of twoway kdensity or the ggplot2 package in R with geom_density or SAS densityplot.

## Recommendations for multiple continuous outcomes

### Scatterplot matrix 

#### Plot description

Multiple scatterplots of continuous outcomes arranged in a matrix, each display the relationship between values at two different time points—for example, baseline values along the horizontal axis and the participant’s maximum value over follow-up along the vertical axis (fig 1, supplement 2 fig A10). The dashed lines represent the boundary between normal and abnormal thresholds.

#### Implementation and interpretation

In our example, where a higher threshold is worse, participants of most concern would be in the top left quadrant (ie, participants’ baseline values were normal and are now abnormal) and the participants who have improved would be in the bottom right (ie, participants’ baseline values were abnormal and are now normal). If more participants from the intervention group than control group were in the top left quadrant this would be cause for concern. In figure 1, slightly more participants in the placebo group (n=4) had higher alanine transaminase (ALTs) when receiving treatment compared with baseline in contrast with participants in the mepolizumab group (n=2).

#### Recommendation

The scatterplot matrix is recommended in an exploratory setting to identify any outliers or patterns of interest. We suggest labelling outlying values with a participant identifier, as shown in figure 1, to assess if one or more participants have abnormal measurements across outcomes. This could be useful to monitor participants in ongoing studies and might also help to raise signals for potential adverse drug reactions in final analyses.

#### Possible adaptations

This plot could be used to explore two continuous measures at any time point over study follow-up. Variations in symbol style and colours should be used to help separate overlapping measurements between groups. Reference lines could be included to indicate both upper and lower limits of normal for each outcome.

#### Limitations

This plot presents several visual problems. Use of solid colours results in occlusion, making it difficult to distinguish individual points; transparency options could help with this issue.

#### Software

Scatterplots are easily implemented as standard plots across the variety of statistical packages. For example, use of twoway scatter in Stata to produce the individual plots and the graph combine command or use of the grc1leg command to produce the scatterplot matrix.

#### Areas for further development

Among the visualisations considered for displaying multiple time-to-event outcomes, the options available were judged to be poor. Although multiple Kaplan-Meier plots could be used to display information on a limited number of prespecified events of interest, a gap remains in how to visualise multiple time-to-event outcomes simultaneously on the same plot. We discussed the development of novel plots in this setting and we will pursue this in future work.

## Discussion

Randomised controlled trials provide a valuable source of data to compare harm outcomes between treatment groups and can help to identify potential signals for adverse drug reactions. However, evidence suggests that practices of reporting data for harm outcomes in clinical trial manuscripts are suboptimal. The CONSORT harms extension^5^ aimed to improve reporting, and the recommendations from Lineberry et al^6^ provided detailed examples to be used alongside the CONSORT harms extension. Both recommendations called for use of visualisations when reporting harm outcomes but did not give guidance on what visualisations would be helpful. Researchers have called for information on appropriate methods of analysing and presenting harm outcomes and for case studies detailing examples of use.^12^

### Principal findings

Our aim was to provide consensus recommendations developed over a series of virtual meetings with researchers responsible for producing clinical trial manuscripts, including clinical trial statisticians and researchers from both academia and industry, as well as clinicians. We have provided examples of the endorsed visualisations to communicate risks of harms in the randomised controlled trial setting that can be used as an alternative to the widely used contingency tables. Our purpose was to increase the use of visualisations for harm outcomes in clinical trial manuscripts and reports and promote presentation of clearer and more informative information on harm outcomes to aid interpretation. Each of the endorsed visualisations can be constructed in standard statistical software and we have signposted to accessible code, when available, for implementation, with the aim of supporting adoption and to ensure efficient application of the recommendations. Trialists can implement our recommendations alongside the CONSORT harms extension^5^ and the recommendations of Lineberry et al,^6^ as well as the more general guidance on the content of statistical analysis plans from Gamble et al.^26^

The choice of visualisation will depend on the outcome type (eg, binary, count, time-to-event, or continuous), the scenario (eg, summarising multiple emerging events or one event of interest), the trial design (trials with >2 treatment groups require more care), and the purpose of the plot (eg, to communicate information about the entire harm profile or to convey a direct message about a particular event of interest). It is for the statistician and clinical trial team to decide the most appropriate visualisation or visualisations for their data and objectives. A combination of plots is likely to be necessary—for example, presenting the traditional Kaplan-Meier plot alongside the survival ratio plot for prespecified harm outcomes to explore the temporal relationship, in addition to the dot plot to summarise the overall harm profile. Researchers can use the decision tree (fig 2) to support their choices, but this tool is not suitable for all circumstances; consideration is still required when deciding the most appropriate visualisations. Different metrics will need to be used depending on what is important to show. For example, for continuous outcomes some of the plots include the standard deviation, which measures the amount of variability of individual data from the sample mean, some include the standard error, which is a measure of precision of the sample mean, and others include the 95% confidence interval, which is 1.96 multiplied by the standard error. In these examples, we have presented what was originally proposed, with context usually dictating the most suitable metric, which will be guided by the purpose of the plot.

Although these recommendations give a clear steer on the type of visualisations to consider, with some guiding principles on format, users can vary many aspects of plot design. For example, colours and symbols used, axis scales and limits, text formatting, appropriate use of labels, and number of groups being compared at once can all affect interpretation and understanding. Much has been written on these aspects, and we refer readers to the articles by Unwin and Muth,^15^ ^27^ as well as lists of key principles for a good visualisation in several publications.^1^ ^28^ ^29^

### Strengths and limitations of this work

The predominance of statisticians over other researchers in the consensus group could be deemed a limitation of this work. Statisticians are, however, typically responsible for producing information on harms, such as in tables or visualisations, and thus the implementation of these recommendations. We therefore deemed their inputs and opinions highly relevant to the process. In addition to statisticians, a graphic designer was present across all meetings, and feedback was sought from each continually throughout the project. To ensure breadth of input, we worked with clinicians with experience in clinical trials to seek their feedback on the endorsed plots and to ensure understanding of each plot because they are likely to be the main consumers of such information. This collaboration with clinicians allowed us to incorporate clarifications into the recommendations where necessary. We hope that choosing clinicians who are active trialists will help to assist with dissemination of our findings and help us to increase the likelihood of these plots being used in practice. Patients were not involved in this work because our focus was to identify the best plots to present in scientific journals with a predominantly scientific readership. Our aim was to first provide guidance and tools to the authors of reports of randomised controlled trials. The next step that needs to be addressed is patient feedback. We did not consider use of interactive visualisations in these recommendations because we believe that these are in their own separate domain and require different considerations for appraisal (see Wang et al^30^). Given the multifaceted, complex nature of data for harms and advances in the way readers consume and access journal articles, interactivity could be highly advantageous for future projects.

Several novel visuals were considered for endorsement in this work (eg, the volcano and tendril plot shown in supplement 3 figs A11 and A15), but the appraisals showed their inadequacies and a preference for more traditional plots. Endorsement was given for two less commonly used plots—the survival ratio plot and the plot of the mean cumulative frequency, and we encourage use of such plots with clear explanations to ease interpretation. We particularly encourage use of the mean cumulative function plot as a summary of the overall burden of harm in place of, or in addition to, summaries of time to discontinuation that are often reported as a proxy for harm. Given the scarcity of visualisations for presenting data for harm outcomes for randomised controlled trials, use of any visualisation of these harms is arguably novel, especially for emerging events. Once the use of visualisations for harm outcomes is more common in scientific publications, the desire for more innovative plots might increase.

Although we suggest amendments to existing plots, the purpose of this work was not to develop new plots. However, it was clear that new approaches are needed for some scenarios, particularly when the visualisation of multiple time-to-event outcomes or multiple continuous outcomes is of interest, or when consideration of duration of events is important. Development of new plots will be undertaken in future work and we will seek to update guidelines to reflect any future progress. With a high likelihood of future updates being required, development of a website that can be more readily updated over time without need for new publications is one further thing to explore and has previously been advocated by Chuang-Stein and Xia.^10^ This would also serve as a readily available resource for dissemination. The CTSpedia Wiki page created by scientists from industry, academia, and the US Food and Drug Administration goes some way towards this, serving as a repository of potential visualisations, although it provides limited direction on benefits of each plot, cautions of use, and possible inferences to be drawn; it has not been updated since 2014.^1^

### Conclusions

Visualisations provide a powerful tool to communicate harms in clinical trials, offering an alternative perspective to the traditional frequency tables. Implementation of the recommendations in this article should improve reporting of harm outcomes in clinical trial manuscripts and enable clearer presentation of harm profiles, and should help to identify potential signals for adverse drug reactions for further monitoring. We endorse each of the plots presented; however, we also highlight the limitations of each plot and provide examples of when their use would be inappropriate. We also caution users to practise care when creating and interpreting each plot. Although the decision tree aids the choice of visualisation, statisticians and clinical trial teams must ultimately decide the most appropriate visualisations for their data and objectives. We recommend trialists continue to examine crude numbers alongside visualisations to fully understand harm profiles. This information should also be reported in supplementary appendices so that readers of trial manuscripts can also appraise this information and so that the data are available to researchers who want to undertake systematic reviews and meta-analyses of harms.^31^

**What is already known on this topic**

Harm outcomes data are complex, but visualisations can provide a clear summary of the harm profile and help identify potential adverse drug reactions
Reporting data for harm outcomes in clinical trial manuscripts can be suboptimal
Researchers have requested guidance on appropriate visualisations for harm outcomes and case studies detailing examples of use

**What this study adds**

To aid researchers in their choice of visualisations, this study undertook a consensus and endorsed visualisations, presented alongside a decision tree, to communicate harms in the randomised controlled trial setting that can be used as alternatives to the widely used contingency tables
The choice of visualisation will depend on outcome type (eg, binary, time-to-event), scenario (eg, summarising multiple emerging events), trial design (trials with >2 treatment groups require more care), and purpose of the plot (eg, to communicate information about the entire harm profile)
Increasing the use of visualisations will provide clearer presentation of information on harm outcomes and thus enable informative interpretation, especially for assessing the harm profile

## Data Availability

The datasets used in this analysis are available from GlaxoSmithKline via ClinicalStudyDataRequest.com, but restrictions apply to the availability of these data, which were used under licence for the current study. The synthetic dataset example is available for download in the Stata aedot and aevolcano command packages.

## Web extra

Extra material supplied by authors

Web appendix: Supplement 1: methodological details

Web appendix: Supplement 2: recommended plots

Web appendix: Supplement 3: Visualisations considered but not recommended

Web appendix: Supplement 4: Example R code and dataset to create the dot plot

Web appendix: Supplement 5: Table and figures summarising initial appraisals of all plots by outcome type

Web appendix: Supplement 6: Tables summarising Mentimeter votes to decide which plots to take forward and amendments

Web appendix: Supplement 7: Free text comments accompanying initial appraisals of recommended plots

## Notes

Contributors: RP and VC conceived and designed this project. RP prepared materials for consensus meetings with feedback provided by VC. RP, VC, and SC designed and facilitated the consensus meetings. RP was responsible for undertaking the interviews with clinicians. All authors were involved in discussions and development of the recommendations. RP wrote the first draft of the manuscript and VC provided initial critical revision. All authors critically reviewed, revised, and approved the manuscript for submission. VC provided supervision of this project. RP is guarantor and attests that all listed authors meet authorship criteria and that no others meeting the criteria have been omitted.

The lead author (RP) affirms that the manuscript is an honest, accurate, and transparent account of the study being reported; that no important aspects of the study have been omitted; and that any discrepancies from the study as planned (and, if relevant, registered) have been explained.

Provenance and peer review: Not commissioned; externally peer reviewed.

Dissemination to participants and related patient and public communities: All parties that participated in the development of these recommendations have been sent a copy of this manuscript and encouraged to share the recommendations within their own research communities. Dissemination to the wider clinical trial community will be undertaken via presentations at relevant conferences and seminars.
