# Supplementary material for: Visualising harms in publications of randomised controlled trials: consensus and recommendations
Source: BMJ. 2022 May 16;377:e068983. doi: 10.1136/bmj-2021-068983 (PMC9108928; doi:10.1136/bmj-2021-068983)
Supplement: Supplementary file 1 — Web appendix: Supplement 1: methodological details [file phir068983.ww1.pdf]

Supplement 1: methodological details

EXAMPLE DATASETS

We produced each of the candidate plots using data from four parallel arm pharmacological RCTs, obtained via the ClinicalStudyDataRequest.com initiative from GlaxoSmithKline (GSK). The first was a two-arm (1:1 allocation) study that evaluated the efficacy of mepolizumab compared to placebo in patients with severe eosinophilic asthma (n=135) (ClinicalTrials.gov number: NCT01691508). The second study investigated mepolizumab in patients with severe uncontrolled refractory asthma comparing two doses of mepolizumab to placebo (1:1:1) (n=576) (ClinicalTrials.gov number: NCT01691521). The third study was a two-arm (2:1) trial examining the efficacy, safety and tolerability of paroxetine compared to placebo in adolescents with unipolar major depression (n=286). The fourth was a two-arm (1:1) trial examining the efficacy and tolerability of paroxetine compared to placebo in paediatric major depression (n=206).[1-4] In addition, a synthetic dataset was created based on a RCT of a novel active treatment for eczema compared to placebo (1:1) in adolescents unresponsive to standard care (n=61) (the synthetic dataset is available for download in the Stata aedot and aevolcano command packages).[5 6]

METHODS

Review

A methodology review performed in March 2018 and updated up until October 2019 identified statistical methods specifically developed to analyse harm outcomes, including the use of visualisations.[7] The review identified over 20 unique methods to visually summarise harm data, including binary AEs and continuous laboratory (e.g. blood tests, culture data) and vital signs (e.g. temperature, blood pressure, electrocardiograms) data.[8-15] These identified visualisations were taken forward for evaluation at the consensus meeting. In addition, alternative visualisations that could be adapted to the harm setting were also considered.[16]

Supplement 1: methodological details

The available graphics were categorised according to the type and number of outcome they support and are presented according to these categories in the following sections. Type of outcomes considered included: binary harm outcomes which includes events such as occurrence of a headache or experiencing nausea, count outcomes i.e. the number of occurrences of an event which could include number of headaches experienced over follow-up, time-to-event outcomes which could include time from treatment exposure to headache and continuous outcomes such as individual results from a blood count. Plots considered were suitable for displaying either single outcomes or multiple simultaneously.

Recommendation development

Consensus meeting

In February 2020, the lead organisers (RP, VC and SC) sent emails to members of the UKCRC CTU Statisticians' Operations Group and personal contacts in academia and industry with a known interest in visualisations, and an advert was placed on the PSI (Statisticians in the Pharmaceutical Industry) visualisation special interest group (SIG) homepage seeking researchers with applied experience of analysing trial data. The emails invited recipients to participate in a consensus meeting to take place over the course of three half-day virtual sessions in July 2020.

Twenty-seven participants were invited to attend in line with the CONSORT group executive recommendations to limit meetings to no more than 30 participants.[17] Twenty-three participants contributed to at least one of the sessions over the course of the three days and are listed in the authorship or acknowledgments. This included 20 statisticians from 15 UKCRC registered CTUs, a health economist based at an academic population health department, one industry statistician, and a data graphics designer who sits on the multimedia team at the BMJ.

Supplement 1: methodological details

A week in advance of the first meeting lead organisers shared with participants the graphics proposed for visually summarising harm data identified in the methodological review, graphics proposed from other settings that could be adapted to the harm setting and any suggested initial adaptations, along with a proposed framework for appraisal. This also included a call for suggestions of any plots that may have been inadvertently omitted.

A draft framework for appraisal was developed in advance taking into consideration work from Ballarini et al. who proposed a framework to assess the properties of graphics for subgroup analysis, principles for producing effective visualisations proposed by Gordon and Finch, and discussions amongst lead organisers regarding the important components to communicate when analysing harm outcomes.[18 19] In the first meeting, the draft criteria to appraise each of the graphics were discussed amongst participants and refined based on feedback and group endorsement. The final criteria are included in table A1. Assessment criteria comprised of eight items related to plot content and presentation including: whether the plot clearly displays an effect size for each event; whether it clearly displays a robust measure of uncertainty; and whether it requires supplementary data presentations. Each item was scored on a scale of 1 to 5. Lower scores indicated negative responses such as 'very unclear' or 'very difficult' or 'strongly disagree'. Two further items related to suitability for use in journal articles or interim analysis reports and whether the plot was suitable for explanatory or exploratory analysis. Exploratory analysis was defined as visualisations suited to data exploration to help identify potential signals for ADRs and explanatory analysis was defined as visualisation suited to communicate a message about the data. Participants were also asked to rank each plot in order of preference in relation to other plots within the same category.

Supplement 1: methodological details

Over the course of the first two meetings, each of the graphics and a summary of its main elements were presented in turn by category and discussed. Participants were encouraged to use the audio and the chat function to: raise any queries they had regarding each plot; highlight what they liked or disliked about it; consider in which research contexts they thought it might be useful; and raise any potential problems or opportunities for causing confusion. Participants completed their appraisals for each graphic and were encouraged to include free text comments if they endorsed any of the specific recommendations or adaptations discussed.

Appraisals were returned to the lead organisers following each meeting and results were summarised and shared with participants in advance of the next meeting to inform discussions (summary scores are presented in supplement 5 tables A.2-A.7 and figures A.29-A.34). After examining the initial appraisal results, participants were encouraged to champion low-scoring plots if they felt strongly that they were under-scored and asked to consider where possible adaptations might be needed. Once discussions were concluded, participants voted on whether to take plots forward for further discussion around recommendations for use and refinements. Results of these votes were presented back to the group in real time. If a plot received at least 60% of the available votes, we considered the plot to be endorsed. Scores marginally below this threshold (50-60%) were revisited for further discussions and votes retaken until a consensus could be reached (results summarised in supplement 6 tables A.8-A.14).

For each of the endorsed plots, we summarised the discussions and free text comments from the appraisal sheets and presented them back to the group (summaries of these comments are included supplement 7). Participants were given the opportunity to raise any other points they felt were important but had been omitted. These included comments about

potential adaptations, where we would recommend using each plot and any cautions or limitations that should be included within the recommendations. We used Mentimeter to record endorsement for each adaptation and finalise the appearance of the endorsed plots and accompanying recommendations.[20] Endorsed plots with incorporated adaptations are presented in the following.

One-to-one interviews with clinicians

In August 2020, two clinical collaborators who are experienced clinical trialists participated in one-to-one semi-structured interviews with one of the lead organisers (RP) lasting approximately one hour. During the interviews, work to date was outlined and feedback on the consensus group's recommendations was sought. This was an opportunity to gather insights on clinicians understanding of the utility and interpretation of each plot, which could then be used to structure the explanatory information provided in the recommendations. Topics covered included:

a. Opinions on the finalised plots, including the merits of each, and whether they were likely to use/endorse these plots in practice;
b. Whether they thought any of the plots were unclear and/or required further explanation that could be incorporated into the recommendations;
c. Whether they thought any modifications were required to any of the plots.

Questions asked were open-ended to allow for detailed responses and comments. We also encouraged clinical participants to raise any other comments they had that were not prompted from the topic areas and invited them to provide written feedback following the meeting if they wished to. Pertinent comments raised were incorporated into the recommendations. Both clinicians are listed as co-authors (AG and NV).

Supplement 1: methodological details

Table A.1: Framework for assessing the properties of graphical displays

Item	Criterion for appraisal	Response options
1	Effect size - Does it clearly display an effect size for events?	1: no/very unclear, 2: unclear, 3: unsure, 4: clear, 5: yes/very clear
2	Treatment effect - Does it clearly display the direction of the treatment effect?	1: no/very unclear, 2: unclear, 3: unsure, 4: clear, 5: yes/very clear
3	Uncertainty – Does it clearly display a robust measure of uncertainty such as CI or SEs?	1: no/very unclear, 2: unclear, 3: unsure, 4: clear, 5: yes/very clear
4	Does it require supplementary data presentations? i.e. Does it stand-alone or does it need additional data presented alongside it?	1: yes/extremely likely, 2: likely, 3: neutral, 4: unlikely, 5: extremely unlikely/stand-alone
5	Can you understand the plot without a detailed explanation?	1: very difficult, 2: difficult, 3: neutral, 4: easy, 5: very easy
6	Do you think non-statistical colleagues i.e. clinicians can understand the plot without a detailed explanation?	1: very difficult, 2: difficult, 3: neutral, 4: easy, 5: very easy
7	How adaptable is the plot for multi-arms/adaptive trials?	1: very difficult, 2: difficult, 3: neutral, 4: easy, 5: very easy
8	Are there limitations around the number of events displayed?	1: yes/extremely limited, 2: very limited, 3: moderately limited, 4: slightly limited, 5: not at all/unlimited
	**Total for items 1-7***	
9	Is it suitable for inclusion in a:	
i	Journal article	1: strongly disagree, 2: disagree, 3: neutral, 4: agree, 5: strongly agree
ii	Final study report	1: strongly disagree, 2: disagree, 3: neutral, 4: agree, 5: strongly agree
iii	Interim analysis report	1: strongly disagree, 2: disagree, 3: neutral, 4: agree, 5: strongly agree
10	Is it best suited to†:	
i	Exploratory analysis	1: strongly disagree, 2: disagree, 3: neutral, 4: agree, 5: strongly agree
ii	Explanatory analysis	1: strongly disagree, 2: disagree, 3: neutral, 4: agree, 5: strongly agree
11	Ranking	1 - most preferred through to least preferred within category
12	Comments: Please indicate if you support any of the amendments proposed for this plot or any other comments on this plot that are not captured elsewhere	Please provide details of the amendment in case of multiple suggested amendments

Abbreviations: CI - confidence interval; SE – standard error

*Total score is a sum of scores assigned to questions 1 through to 7. Scores to question eight were not included, whilst we thought this was important for consideration, we did not wish to disadvantage plots that could only present a limited number of events, as this might be through design and in fact in some settings is likely to be an advantage. The group discussed this point before the decision was made.

† Exploratory analysis was defined as visualisations suited to data exploration to help identify potential signals for adverse (drug) reactions (A(D)Rs) and explanatory analysis was defined as visualisation suited to communicate a message about the data.

Supplement 1: methodological details

References

1. Bel EH, Wenzel SE, Thompson PJ, et al. Oral Glucocorticoid-Sparing Effect of Mepolizumab in Eosinophilic Asthma. *New England Journal of Medicine* 2014;371(13):1189-97. doi: 10.1056/NEJMoa1403291
2. Berard R, Fong R, Carpenter DJ, et al. An international, multicenter, placebo-controlled trial of paroxetine in adolescents with major depressive disorder. *J Child Adolesc Psychopharmacol* 2006;16(1-2):59-75. doi: 10.1089/cap.2006.16.59 [published Online First: 2006/03/24]
3. Ortega HG, Liu MC, Pavord ID, et al. Mepolizumab Treatment in Patients with Severe Eosinophilic Asthma. *New England Journal of Medicine* 2014;371(13):1198-207. doi: 10.1056/NEJMoa1403290
4. Emslie GJ, Wagner KD, Kutcher S, et al. Paroxetine Treatment in Children and Adolescents With Major Depressive Disorder: A Randomized, Multicenter, Double-Blind, Placebo-Controlled Trial. *Journal of the American Academy of Child & Adolescent Psychiatry* 2006;45(6):709-19. doi: https://doi.org/10.1097/01.chi.0000214189.73240.63
5. AEDOT: Stata module to produce dot plot for adverse event data [program]: Boston College Department of Economics, 2020.
6. AEVOLCANO: Stata module to produce volcano plot for adverse event data [program]: Boston College Department of Economics, 2020.
7. Phillips R, Sauzet O, Cornelius V. Statistical methods for the analysis of adverse event data in randomised controlled trials: a scoping review and taxonomy. *BMC Medical Research Methodology* 2020;20(1):288. doi: 10.1186/s12874-020-01167-9
8. Zink RC, Wolfinger RD, Mann G. Summarizing the incidence of adverse events using volcano plots and time intervals. *Clinical Trials* 2013;10(3):398-406.
9. Amit O, Heiberger RM, Lane PW. Graphical approaches to the analysis of safety data from clinical trials. *Pharmaceutical Statistics* 2008;7(1):20-35.
10. Chuang-Stein C, Xia HA. The practice of pre-marketing safety assessment in drug development. *Journal of Biopharmaceutical Statistics* 2013;23(1):3-25. doi: 10.1080/10543406.2013.736805
11. Chuang-Stein C, Le V, Chen W. Recent Advancements in the Analysis and Presentation of Safety Data. *Drug Information Journal* 2001;35(2):377-97. doi: 10.1177/009286150103500207
12. Southworth H. Detecting outliers in multivariate laboratory data. *Journal of Biopharmaceutical Statistics* 2008;18(6):1178-83.
13. Trost DC, Freston JW. Vector Analysis to Detect Hepatotoxicity Signals in Drug Development. *Therapeutic Innovation & Regulatory Science* 2008;42(1):27-34. doi: 10.1177/009286150804200106
14. Karpefors M, Weatherall J. The Tendril Plot—a novel visual summary of the incidence, significance and temporal aspects of adverse events in clinical trials. *Journal of the American Medical Informatics Association* 2018;25(8):1069-73. doi: 10.1093/jamia/ocy016
15. Zink RC, Marchenko O, Sanchez-Kam M, et al. Sources of Safety Data and Statistical Strategies for Design and Analysis:Clinical Trials. *Therapeutic Innovation & Regulatory Science* 2018;52(2):141-58. doi: 10.1177/2168479017738980
16. Phillips RC, VR.; Cro, S.; Sauzet, O. The use of visual analytics for clinical trial safety outcomes: a methodological review. *Trials Meeting abstracts from the 5th International Clinical Trials Methodology Conference (ICTMC 2019)* 2019;20(Supplement 1)
17. Moher D, Schulz KF, Simera I, et al. Guidance for Developers of Health Research Reporting Guidelines. *PLOS Medicine* 2010;7(2):e1000217. doi: 10.1371/journal.pmed.1000217
18. Ballarini NM, Chiu Y-D, König F, et al. A critical review of graphics for subgroup analyses in clinical trials. *Pharmaceutical Statistics* 2020 doi: 10.1002/pst.2012 [published Online First: 25 March 2020]
19. Gordon I, Finch S. Statistician Heal Thyself: Have We Lost the Plot? *Journal of Computational and Graphical Statistics* 2015;24(4):1210-29. doi: 10.1080/10618600.2014.989324

Supplement 1: methodological details

20. Mentimeter [Available from: https://www.mentimeter.com/.
