# Supplementary material for: Visualising harms in publications of randomised controlled trials: consensus and recommendations
Source: BMJ. 2022 May 16;377:e068983. doi: 10.1136/bmj-2021-068983 (PMC9108928; doi:10.1136/bmj-2021-068983)
Supplement: Supplementary file 2 — Web appendix: Supplement 2: recommended plots [file phir068983.ww2.pdf]

Supplement 2: recommended plots

Figure A.1: Dot plot of events - data taken from the two-arm example dataset with 1:1 allocation ratio

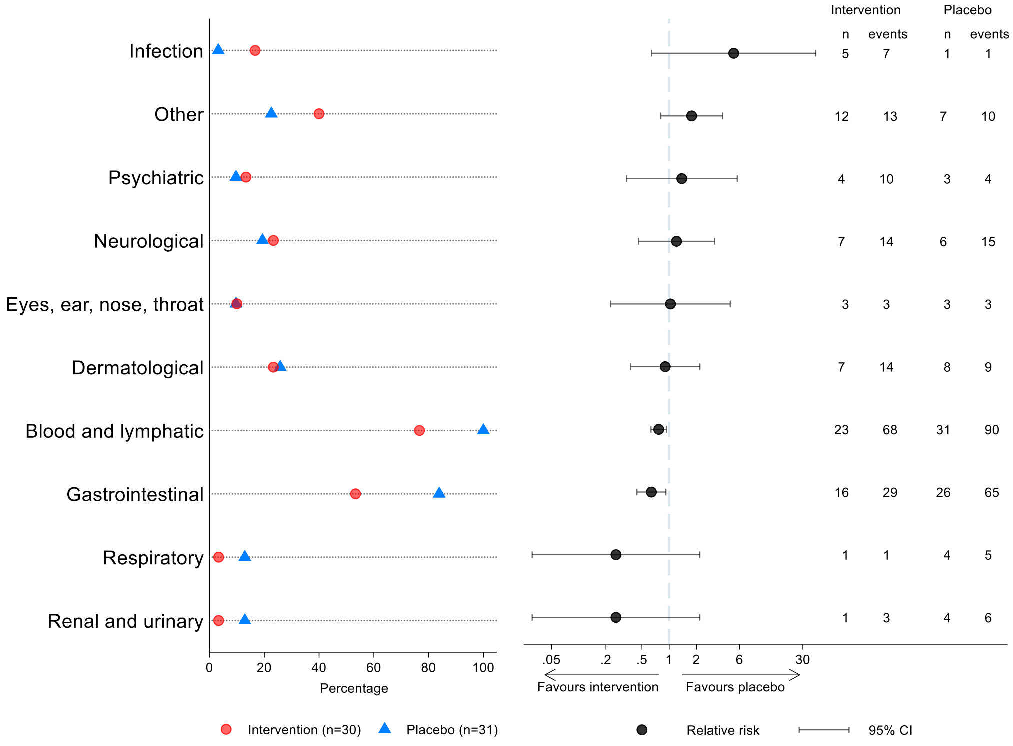

Legend: Dot Plot for emerging harm outcomes between two treatment groups for the simulated dataset. The left panel of the figure displays the percentage of participants experiencing an event (labelled on the y-axis) in the intervention group with a red circle and placebo group with a blue triangle. The central panel displays the relative risk and corresponding 95% confidence interval on the log10 scale and a line to show the value of no difference (for relative risks, this is 1). The right panel displays the 'number of participants experiencing the event at least once' (n) and 'the number of events' (events) (accounting for recurrent events within participants) by treatment group. The dot plot provides a comprehensive visual representation of the entire harm profile.

Supplement 2: recommended plots

Figure A.2: Horizontal stacked bar chart of events by maximum severity – data taken from the two-arm example dataset with 1:1 allocation ratio

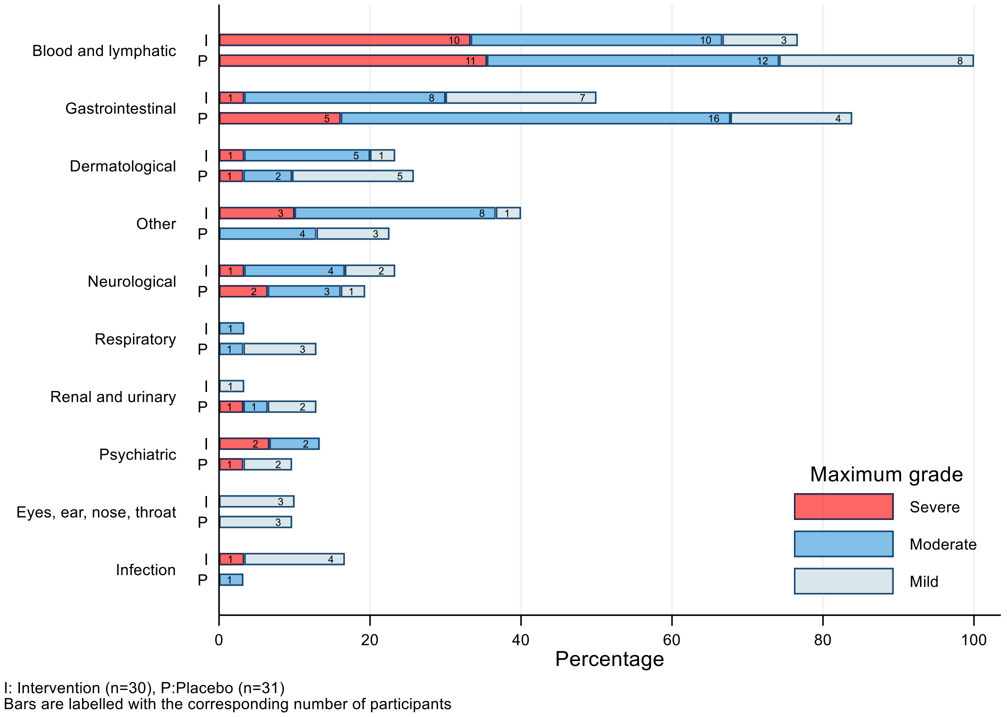

Legend: Horizontal stacked bar chart for emerging harm outcomes by maximum severity and treatment group for the simulated dataset. Total bar height represents the proportion of participants with that event at least once and each bar is split into segments to indicate numbers by severity grading. Bar segments are labelled with the corresponding number of participants. The stacked bar chart used in this way is helpful when it is important to present information on the severity of multiple events.

Supplement 2: recommended plots

Figure A.3a: Bar chart of event counts – data taken from the two-arm example dataset with 1:1 allocation ratio

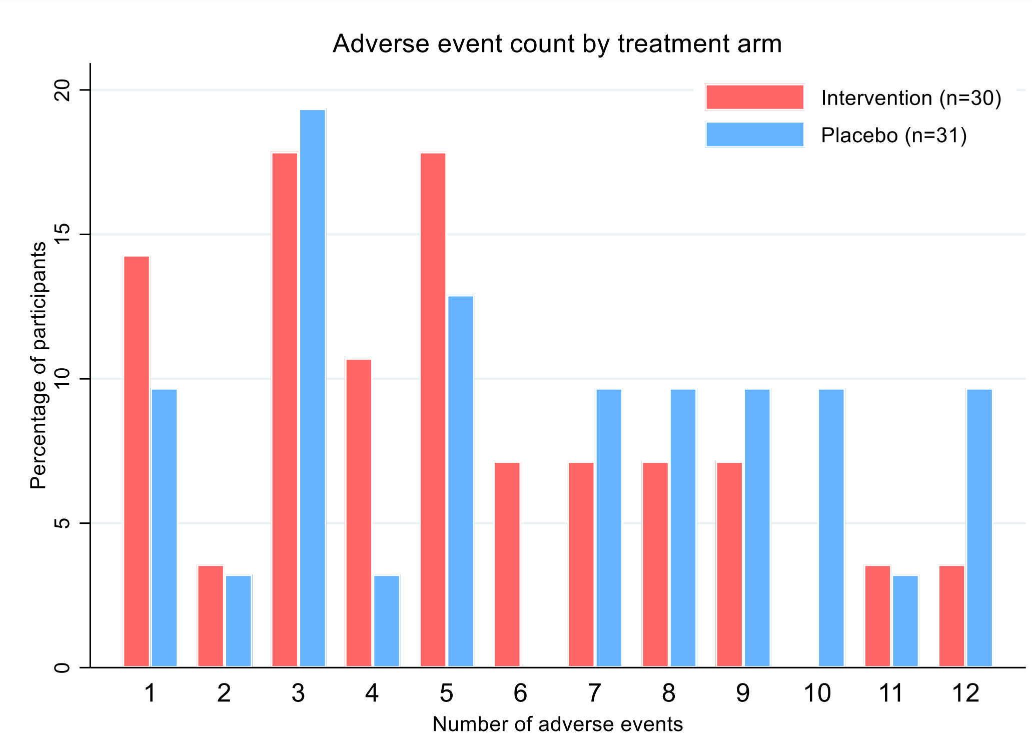

Legend: Bar chart of counts of harm outcomes by treatment group for simulated dataset. Each bar represents the proportion of participants with 0, 1, 2 etc. events for each treatment group. This plot groups all adverse events together. Alternatively, it can be used to summarise this information for specific events of interest. Using the bar chart to present this information can help highlight between group differences in the burden of harm experienced by participants.

Supplement 2: recommended plots

Figure A.3b: Bar chart of event counts – data taken from the three-arm Mepolizumab dataset with 1:1 allocation ratio

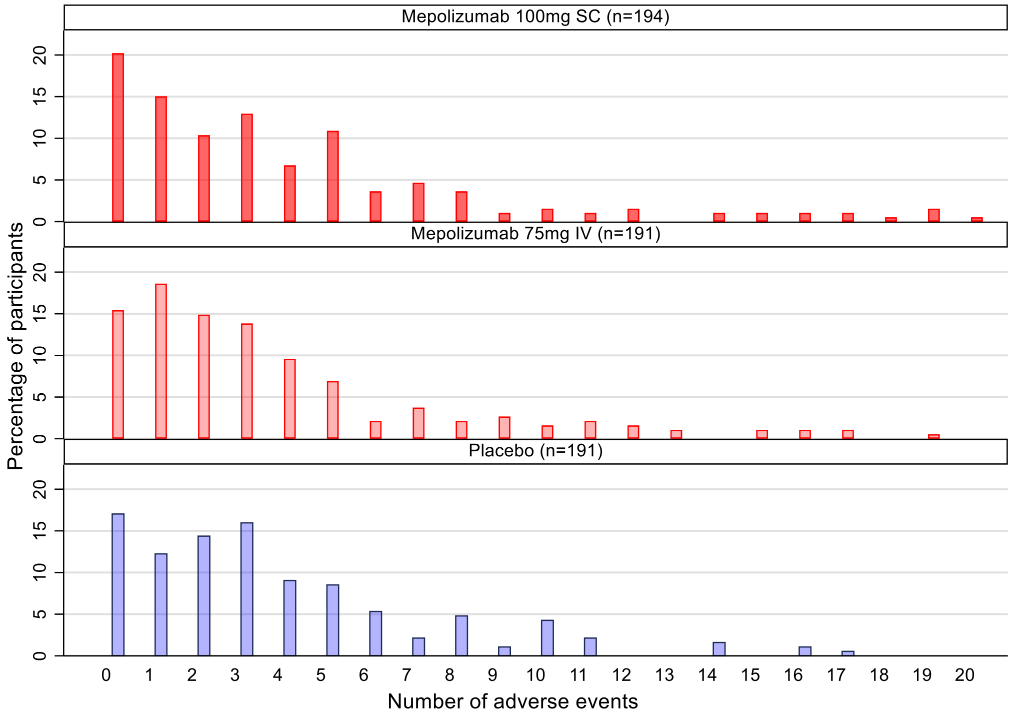

Legend: Bar chart of counts of harm outcomes by treatment group (when > 2 treatment groups). Each bar represents the proportion of participants with 0, 1, 2 etc. events for each treatment group. We recommend separate stacked plots like this for trials with more than two treatment group. Using the bar chart to present this information can help highlight between group differences in the burden of harm experienced by participants.

Supplement 2: recommended plots

Figure A.4: Kaplan–Meier plot for an event of interest – data taken from the two-arm Mepolizumab dataset with 1:1 allocation ratio

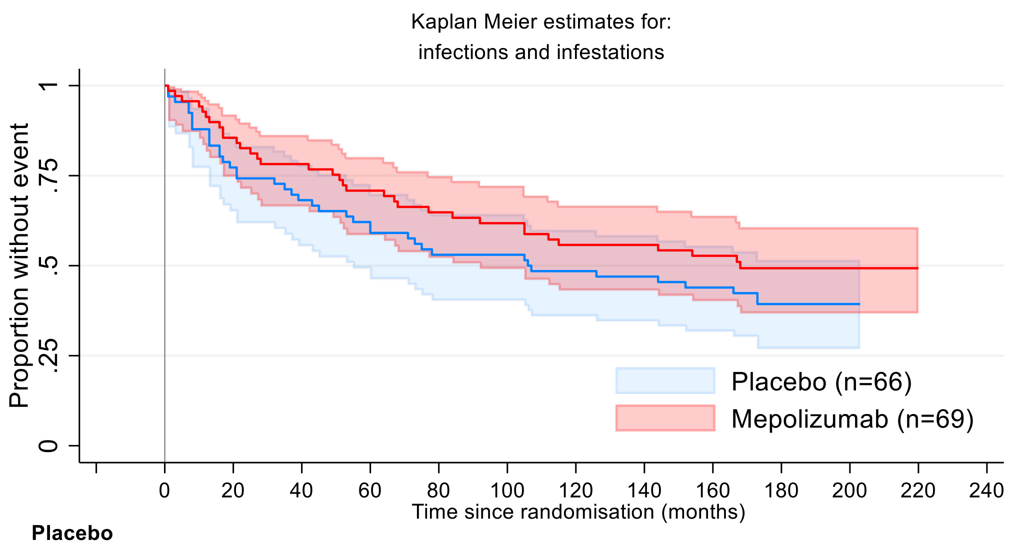

	0	20	40	60	80	100	120	140	160	180	200	220	240
**Placebo**													
At-risk	66	51	45	41	35	35	32	31	29	5	1	0	0
Censored	0	0	0	0	0	0	0	0	0	22	26	27	27
Event	0	15	21	25	31	31	34	35	37	39	39	39	39
**Mepolizumab**													
At-risk	69	59	53	47	43	41	37	37	35	2	1	1	0
Censored	0	0	1	2	2	2	2	2	2	33	34	34	35
Event	0	10	15	20	24	26	30	30	32	34	34	34	34

Legend: Kaplan-Meier plot with an extended at risk table for specific harm outcome of interest by treatment group for the two-arm Mepolizumab study. Plots the survival estimates by treatment group where each line indicates the cumulative proportion of participants remaining event free over time by treatment group with 95% confidence intervals for each group separately. The extended at risk table includes information on the number of participants that remain ‘at risk’, the cumulative number that have been censored and the cumulative number that have experienced an event at discrete time points. In the harm setting, Kaplan-Meier plots can be used to present information for specific events of interest as a useful way to detect a potential disproportionality between treatment groups, which is useful when trying to identify signals for adverse (drug) reactions (A(D)Rs).

Supplement 2: recommended plots

Figure A.5: Mean cumulative function plot for all events – data taken from the two-arm Paroxetine dataset with 1:1 allocation ratio

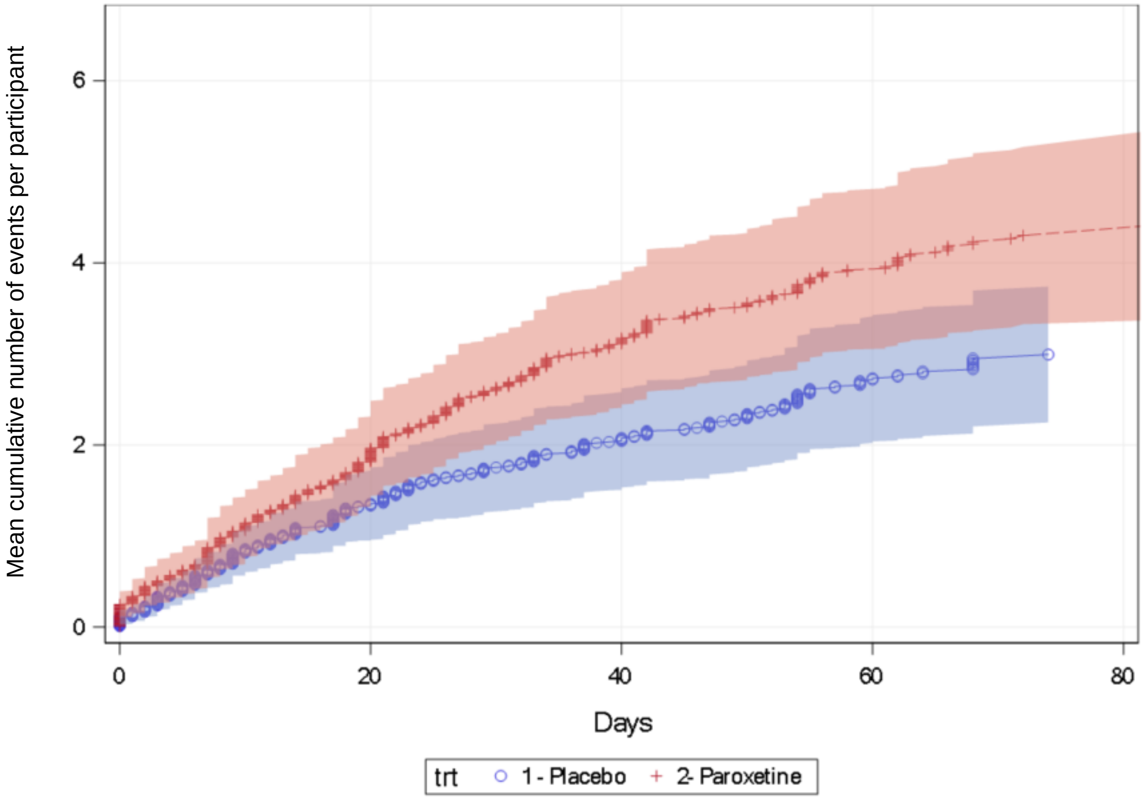

**Placebo**					
At risk	102	98	91	62	12
**Paroxetine**					
At risk	101	95	82	60	13

Legend: Mean cumulative function plot for harm outcomes by treatment group for the Paroxetine study with 1:1 treatment allocation. Plots the mean number of events per participant over time by treatment group and includes 95% confidence intervals within groups. The risk table includes information on the number of participants that remain ‘at risk’ at discrete time points throughout the study. In the harm setting, MCF plots can be used to demonstrate a comparison of the burden of experiencing ‘any event’ or the recurrence of events of special interest.

Supplement 2: recommended plots

Figure A.6: Survival ratio plot for event of interest – data taken from the two-arm Mepolizumab dataset with 1:1 allocation ratio

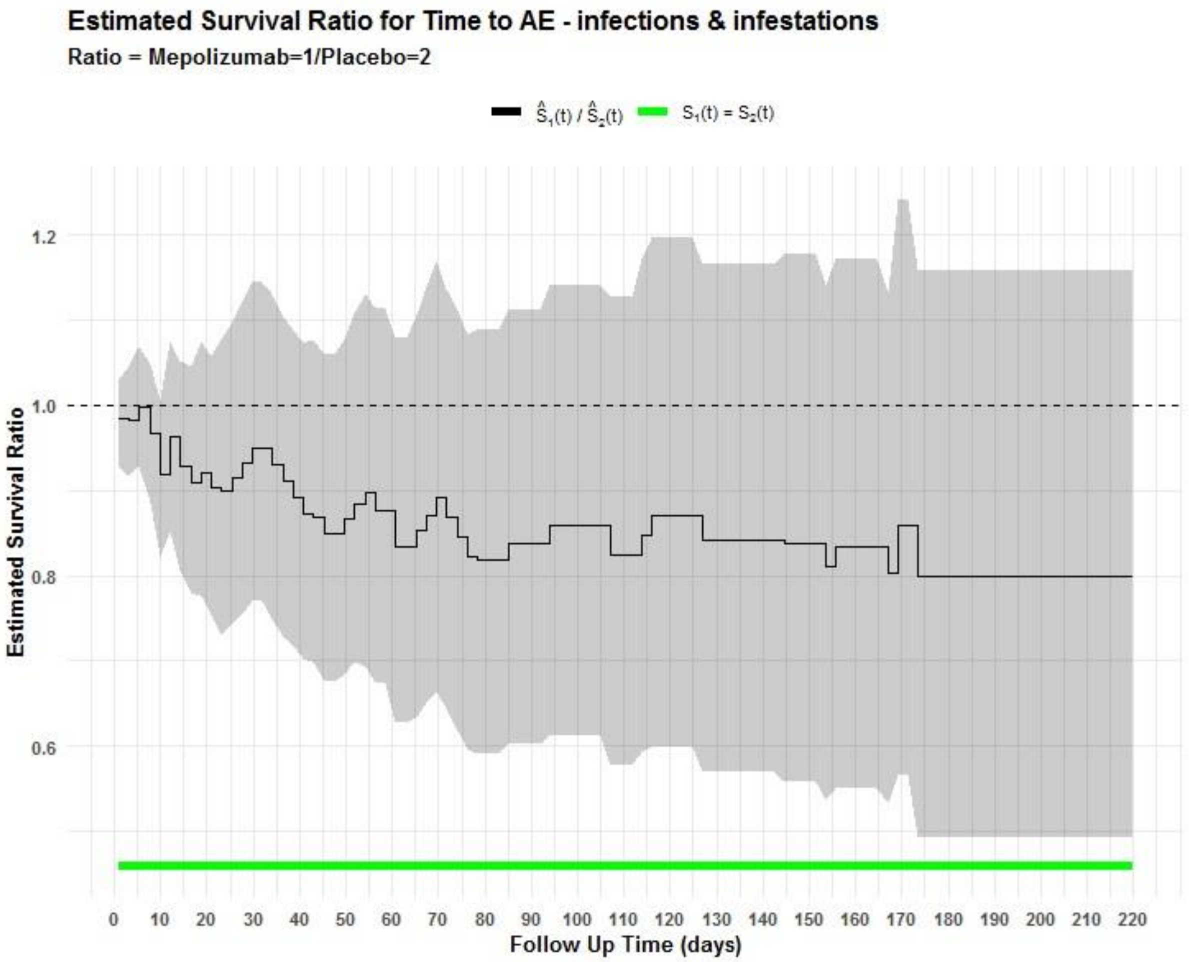

Legend: Survival ratio plot for specific harms of interest for two-arm Mepolizumab study. Plots the ratio of survival estimates (solid black line) with the 95% pointwise confidence bands (grey shaded area), where the dashed line at y=1 represents the line of no difference. Departures from unity are indicated using the horizontal band at the bottom of the plot which is green when confidence band includes 1 and red when excludes 1. In the harm setting, the survival ratio plot would be suitable for signal detection analysis across the body of emerging events, as it provides a between group comparison that can be used to detect departures from unity and help identify the time that such divergences occur, which can help detect potential signals for ADRs.

Supplement 2: recommended plots

Figure A.7: Line graph of summary statistic over time by treatment arm for a continuous harm outcome of interest – data taken from the two-arm Mepolizumab dataset with 1:1 allocation ratio

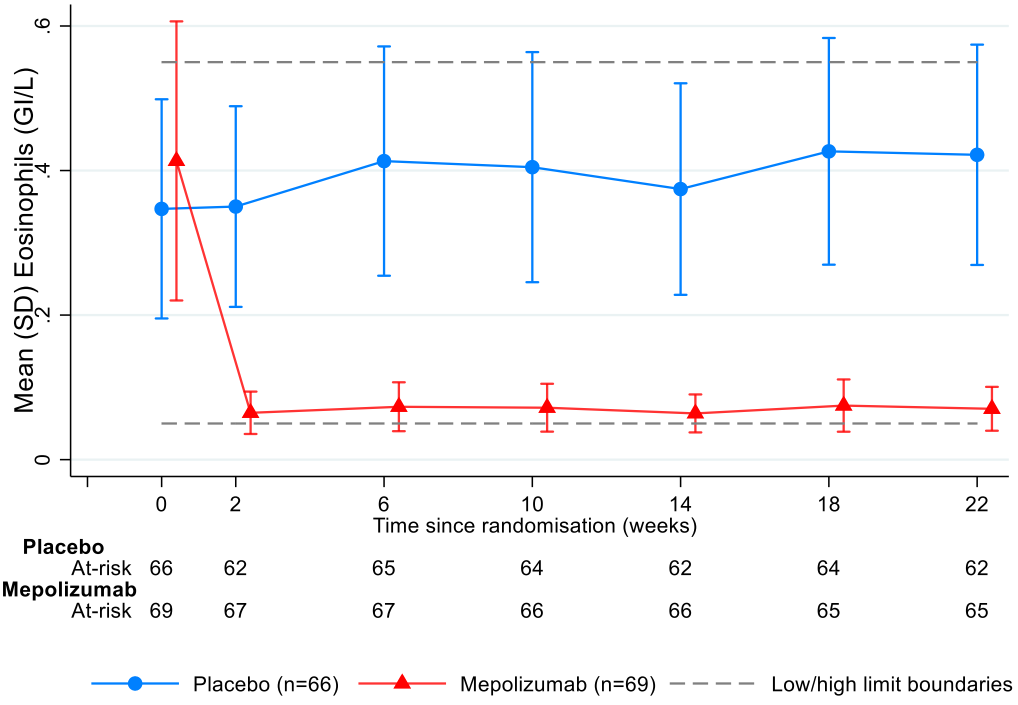

Legend: Line graph with risk table for specific continuous outcome of interest by treatment group over time. The markers display an appropriate summary statistic (in this example the mean) and the vertical lines indicate a measure of variability (in this example the standard deviation) of raw values at each discrete point, connected with a line for each treatment group. This plot can be used to describe continuous harm outcomes of interest over time and can help identify shifts in distributions between treatment groups.

Supplement 2: recommended plots

Figure A.8: Violin plot summarising the distribution of a continuous harm outcome of interest over time – data taken from the two-arm Mepolizumab dataset with 1:1 allocation ratio

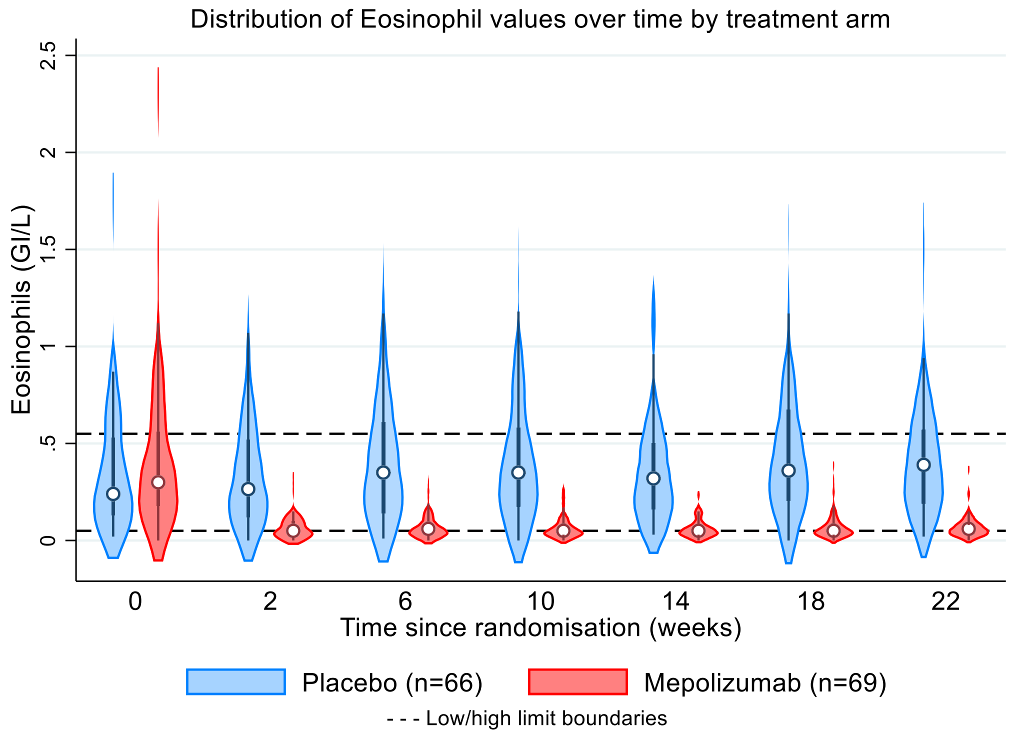

Legend: Violin plot for specific continuous event of interest by treatment group over time. The hollow circle markers indicate the median, the boxes indicate the inter-quartile range and the lines extend to minimum and maximum points, overlaid with kernel density plots. The violin plot is a useful alternative to the line graph when presenting a continuous outcome that is far from a normal distribution and/or the user is interested in exploring the distribution. It can also help identify outliers and/or identify participants who are persistently showing values of concern.

Supplement 2: recommended plots

Figure A.9: Kernel density plot for a continuous harm outcome of interest - data taken from the two-arm Paroxetine study with 2:1 allocation ratio

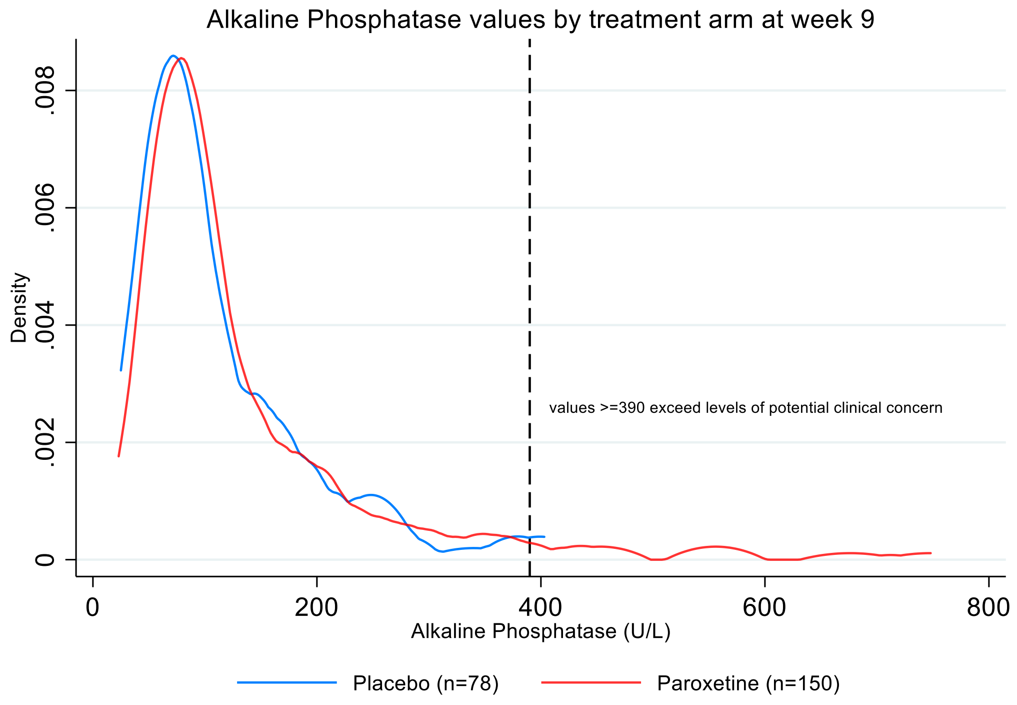

Legend: Kernel density plot for a specific continuous outcome of interest by treatment group, at a single time point, with a reference line to indicate values above which are of clinical concern. It can be helpful to identify shifts in distributions between treatment groups.

Supplement 2: recommended plots

Figure A.10: Scatterplot matrix for continuous harm outcomes – data taken from the two-arm Mepolizumab dataset with 1:1 allocation ratio

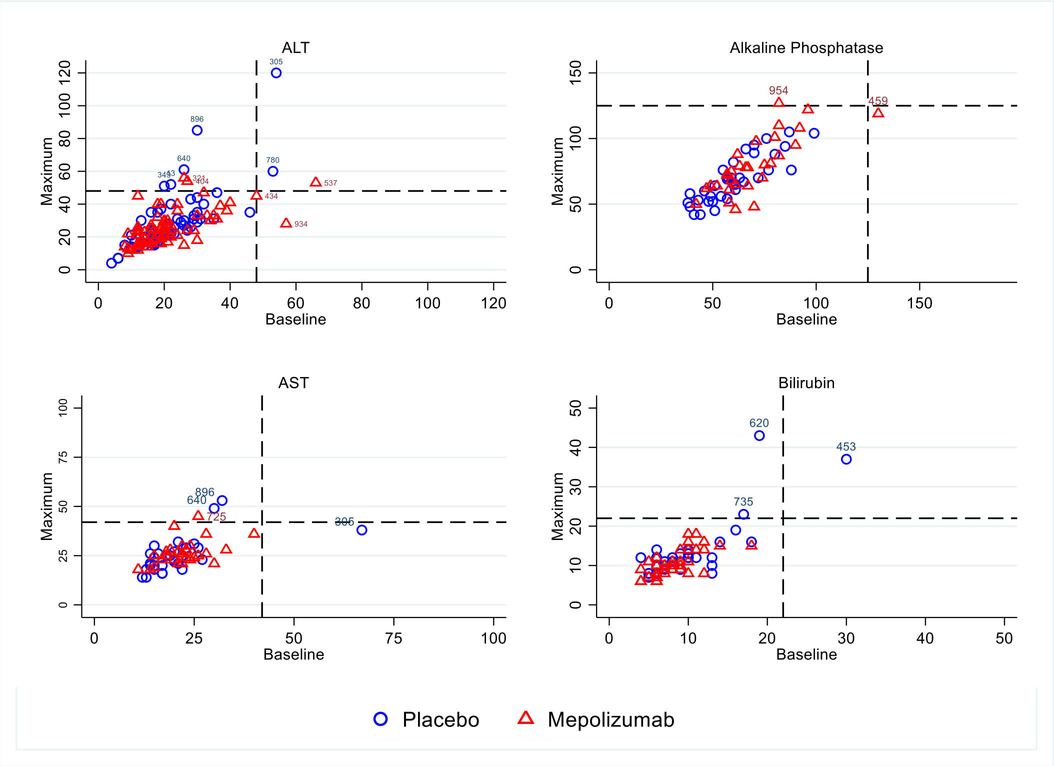

Legend: Scatterplot matrix for multiple continuous harm outcomes by treatment group. Plots each participant’s baseline value against their maximum on-treatment value. The dashed lines represent the boundary between normal and abnormal thresholds. Outlying observations are labelled with participant identification numbers. This plot can be used in an exploratory setting to identify any outlying observations and to help identify any patterns within participants. In this example where a higher threshold is worse, participants of most concern would be in the top left quadrant (i.e. participants’ baseline values were normal and are now abnormal) and the participants who have improved would be in the bottom right (i.e. participants’ baseline values were abnormal and are now normal).
