# Supplementary material for: Visualising harms in publications of randomised controlled trials: consensus and recommendations
Source: BMJ. 2022 May 16;377:e068983. doi: 10.1136/bmj-2021-068983 (PMC9108928; doi:10.1136/bmj-2021-068983)
Supplement: Supplementary file 3 — Web appendix: Supplement 3: Visualisations considered but not recommended [file phir068983.ww3.pdf]

Supplement 3 - Visualisations considered but not recommended

Figure A.11: Volcano plot

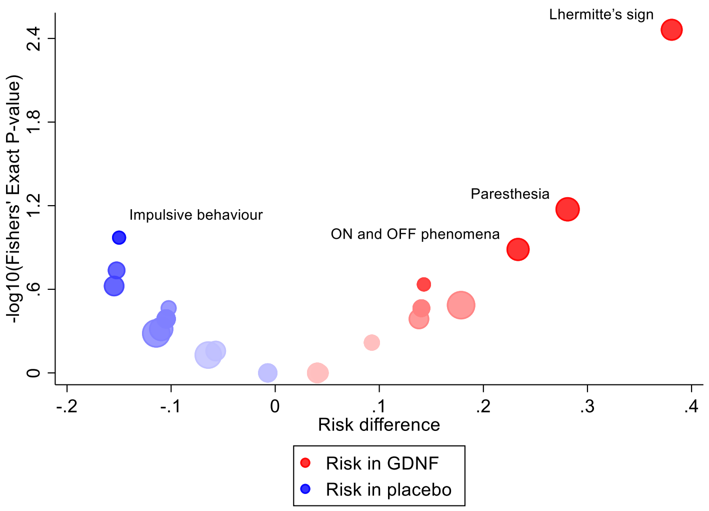

Figure description: Each bubble/circle represents a distinct event. Bubble size/area is proportional to the total number of events across treatment arms. The x-axis indicates the size of the treatment effect. The colour of the bubbles is used to indicate the direction of the treatment effect. The y-axis is used to display log transformed p-values. The colour saturation of each bubble corresponds to the size of the p-value. Under the null hypothesis we would expect to see a U-shape curve with a random scatter of events around the null value, in this case a risk-difference of 0. *Original plot first proposed in: Zink RC, Wolfinger RD and Mann G. Summarizing the incidence of adverse events using volcano plots and time intervals. Clinical Trials 2013; 10: 398-406. Data taken from: Whone A, Luz M, Boca M, et al. Randomized trial of intermittent intraputamenal glial cell line-derived neurotrophic factor in Parkinson's disease. Brain 2019; 142: 512-525*

Adaptions considered: The x-axis could be used to display different metrics e.g. risk difference, odds ratio, incident rate ratios. The p-values displayed on the y-axis can be based on any statistical test and can incorporate a multiple test correction. Colour saturation could be used to reflect an alternative to p-value size such as the average severity rating – this has been suggested by multiple people when demonstrating this plot

Supplement 3 - Visualisations considered but not recommended

Figure A.12: Alternative volcano 1 proposed by BMJ graphic designer (WST)

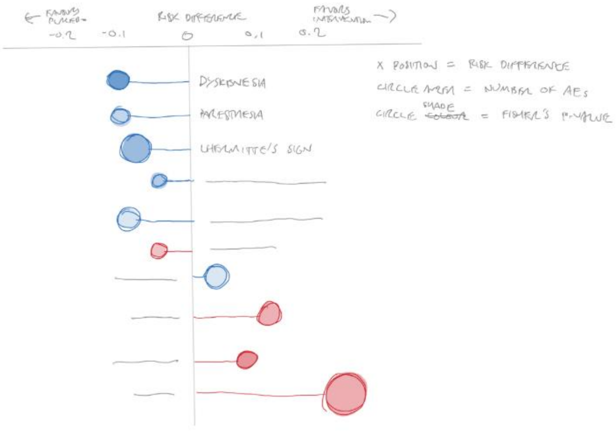

Figure description: Displays the risk difference across the x-axis. The direction of treatment effect is indicated by colour. The size of the p-value is indicated by the colour shade/saturation. Total number of events indicated by the circle area. Allows incorporation of labels for all events.

Adaptions considered: Could incorporate some measure of precision e.g. add 95% CI bars or shade/saturation could reflect standard error instead of the size of the p-value.

Figure A.13: Alternative volcano 2 proposed by BMJ graphic designer (WST)

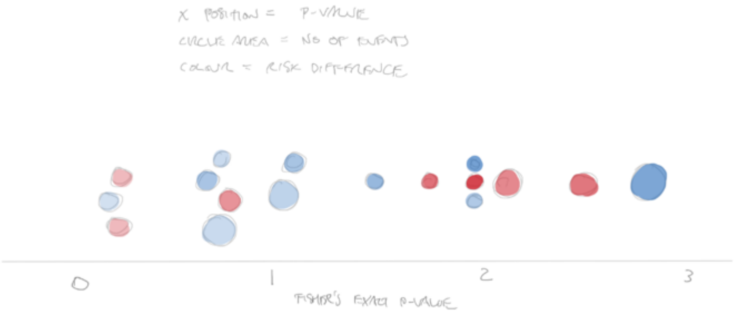

Figure description: The size of the risk difference is reflected in the colour saturation of each circle. The direction of the treatment effect is indicated by the colour of the circles. The size of the p-value is displayed across the x-axis. The total number of events is indicated by the circle area. The y-axis is not used to display a metric but instead used to stack circles/bubbles to prevent overlap.

Adaptions considered: Recommend including a legend/key to indicate which colour represents which treatment arm and size of effect that the colour shades/saturation corresponds to. Not clear how would incorporate labels.

Supplement 3 - Visualisations considered but not recommended

Figure A.14: Alternative volcano 3 proposed by BMJ graphic designer (WST)

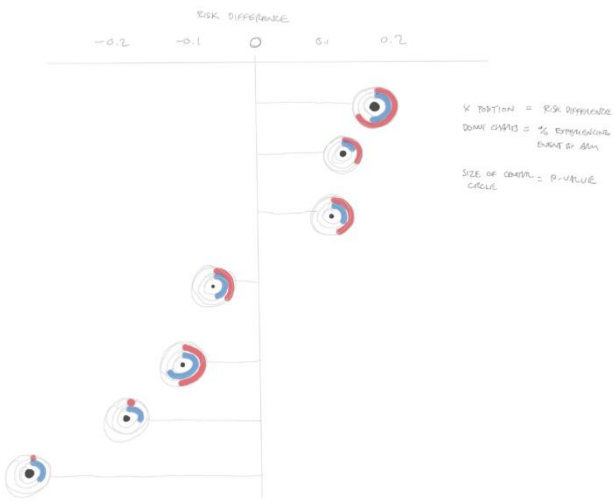

Figure description: Shows size and direction of treatment effect across the x-axis. The size of the p-value is indicated by the size of the central black circle. The number of events is represented by the proportion of outer segments (or donuts) that are shaded, with different colours for each treatment arm. Each event takes up a ‘row’ along the y-axis. BMJ graphics designer (Will Stahl Timmins – WST) proposed this as an idea but suggests needs further refinement (please see comment below).

“If you wanted to actually show the number of people in each arm that experienced events rather than just the sum total that should be possible too. Here's one idea, but not a very good one. Concentric donut charts have issues - the inner ones tend to look smaller than the outer ones. But given time one might come up with a better one. I guess the point is, we need to work out what the most important information to show is, and then work from there…...”

Figure A.15: Tendril plot

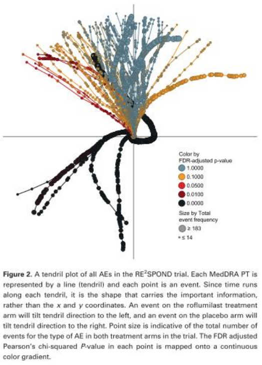

Figure description: Each event term is represented by a line (or tendril). Each point on the line indicates the occurrence of an event. The distance from the origin indicates the time the event occurred. The direction or tilt of the line is used to indicate the treatment arm the event occurred in i.e. the line takes a unit tilt to the left for an event in the intervention arm and a unit tilt to the right for an event in the control arm. The colour of the points along the lines indicate the size of the p-value. Reprinted from *Karpefors, M. and J. Weatherall (2018). "The Tendril Plot—a novel visual summary of the incidence, significance and temporal aspects of adverse events in clinical trials." Journal of the American Medical Informatics Association* ***25****(8): 1069-1073 with permission of Oxford University Press.*

Adaptions considered: This plot was initially proposed interactively so when you hover over a line it shows the event name. If it is to be used as a static plot event labels would need to be incorporated.

Supplement 3 - Visualisations considered but not recommended

Figure A.16: Heat map

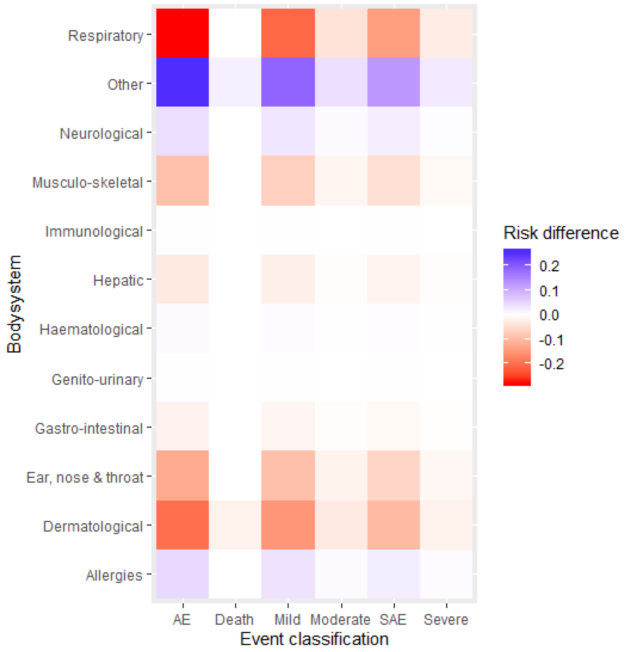

Figure description: Individual event names are displayed along the y-axis. Different event classifications such as severity ratings are displayed across the x-axis. Each x/y-axis grid position/square is coloured to represent a treatment effect for a unique event and classification. The colour of the squares/grid is used to indicate the direction of the (standardised) treatment effect. Colour saturation of the squares/grid is used to indicate size of effect for each AE. *Original plot first proposed in: Zink, R. C., et al. (2018). "Sources of Safety Data and Statistical Strategies for Design and Analysis: Clinical Trials." Therapeutic Innovation & Regulatory Science* ***52****(2): 141-158.*

Adaptions considered: The number of participants with each event/classification could be added to the squares/grid as per the level plot. The proportion of the square/grid coloured for each event/category combination could be used to indicate number of events as per the level plot (displayed on page 14)

Supplement 3 - Visualisations considered but not recommended

Figure A.17a: Level plot - Originally proposed for categories of abnormal blood tests

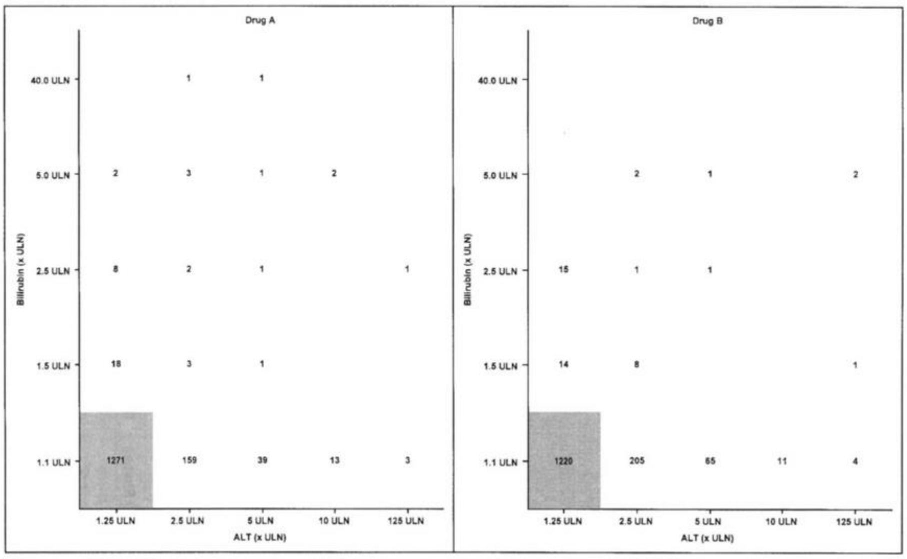

FIGURE 7. A simultaneous grade change in ALT and bilirubin.

Figure description: Displays categories of two different blood tests on the x and y-axes. Displays counts of participants in the intersection of categories. *Reprinted from: Chuang-Stein, C., et al. (2001). "Recent Advancements in the Analysis and Presentation of Safety Data." Drug Information Journal 35(2): 377-397 under the terms of the Creative Commons CC BY License.*

Adaptions considered: An adaption similar to this from Ballarini et al. could provide potentially useful modifications for the heat map. *Reprinted from: Ballarini, NM, Chiu, Y-D, König, F, Posch, M, Jaki, T. A critical review of graphics for subgroup analyses in clinical trials. Pharmaceutical Statistics. 2020; 1– 20. https://doi.org/10.1002/pst.2012 under the terms of the Creative Commons CC BY License.*

Figure A.17b Level plot – taken from Ballarini et al. for consideration of potentially useful modifications for the heat map

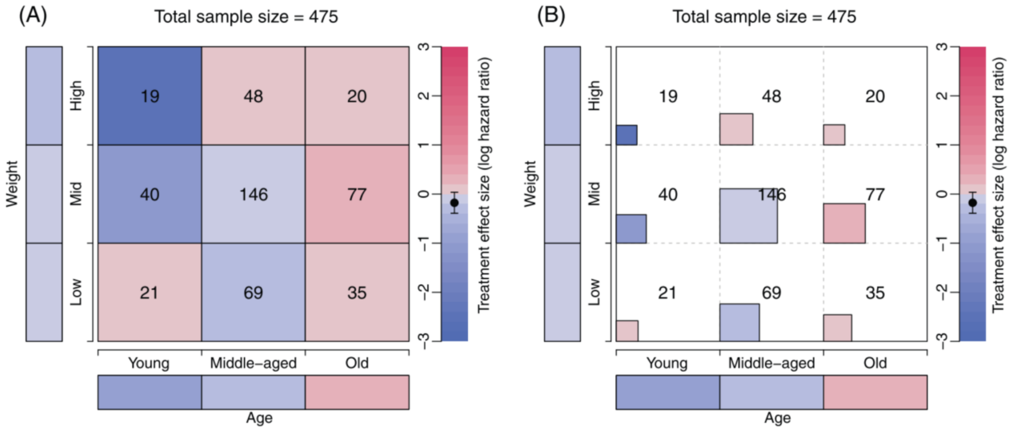

FIGURE A2 Level plots of treatment effect in terms of the log-hazard ratio across mutually disjoint subgroups defined by *age* and *weight* categorised in three levels. The cells on the bottom and the left margins correspond to the marginal subgroups defined by the levels of *age* and *weight*. In B, the area of each square inside the cells is proportional to the sample sizes, which are also displayed in the middle of the cells

Reprinted from: Ballarini, NM, Chiu, Y-D, König, F, Posch, M, Jaki, T. A critical review of graphics for subgroup analyses in clinical trials. Pharmaceutical Statistics. 2020; 1– 20, https://doi.org/10.1002/pst.2012 under the terms of the Creative Commons CC BY License.

Supplement 3 - Visualisations considered but not recommended

The following two figures have not been specifically proposed for the analysis of harm outcomes but were suggested as potentially useful plots by academic colleagues:

Figure A.18: Star plot

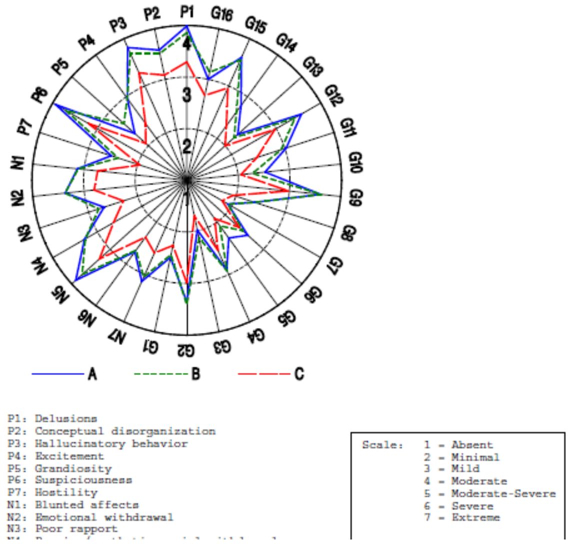

Figure description: Displays mean values for multiple clinical results (in this example each of the 30 PANSS items rated on a Likert scale). The coloured lines represent different treatment arms. Concentric reference lines included to help read off values. Could be a**dapted to** present mean grade for each AE by treatment arm. *Thanks to Steven Julious at Sheffield University for flagging this plot. Reprinted from: Squassante et al. Simple graphical methods of displaying multiple clinical results. Pharmaceut. Statist. 2006; 5: 51–60 with permission from John Wiley & Son*

Adaptions considered: Could present mean grade for each event by treatment arm

Figure A.19: Alluvial plot

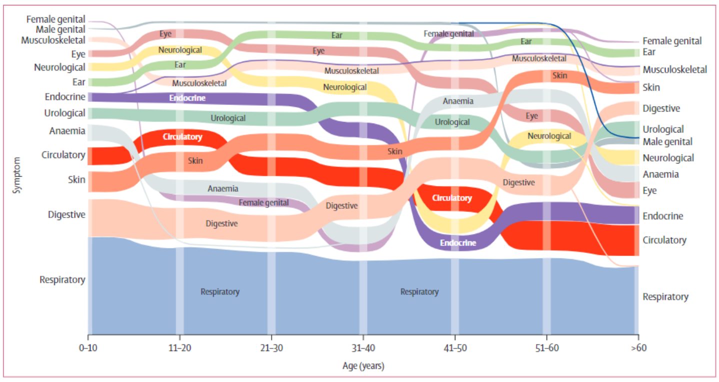

Figure 4: **Alluvial graph of disease or symptom prevalence across decades of life**
Width of bands corresponds to relative proportion of symptoms or medical conditions, with the y axis organised to have the most prevalent conditions closest to the x axis. Associations between symptoms or conditions are represented as offshoots that connect systems. The light shaded vertical bars correspond to the decade-wise groupings used in analysis..

Figure description: Shows how the proportions experiencing an event (for multiple events) change over time. Could be adapted to show changes in severity categories over time for a single event, would be tricky to do this for multiple events. Would need to produce a separate plot for each arm to make a comparison between treatment arms. *Thanks to Marianna Nodale at Cambridge University Hospital for suggesting this image. Reprinted from: Salvi S, Apte K, Madas S, et al. Symptoms and medical conditions in 204 912 patients visiting primary health-care practitioners in India: a 1-day point prevalence study (the POSEIDON study). Lancet Glob Health. 2015;3(12):e776-e784. doi:10.1016/S2214-109X(15)00152-7 under the terms of the Creative Commons CC BY NC ND License*

Adaptions considered: Could be used to show changes in severity categories over time for a single event, would be tricky to do this for multiple events. Would need to produce a separate plot for each treatment group to make a comparison between treatments.

Supplement 3 - Visualisations considered but not recommended

Figure A.20: Histogram of counts over time

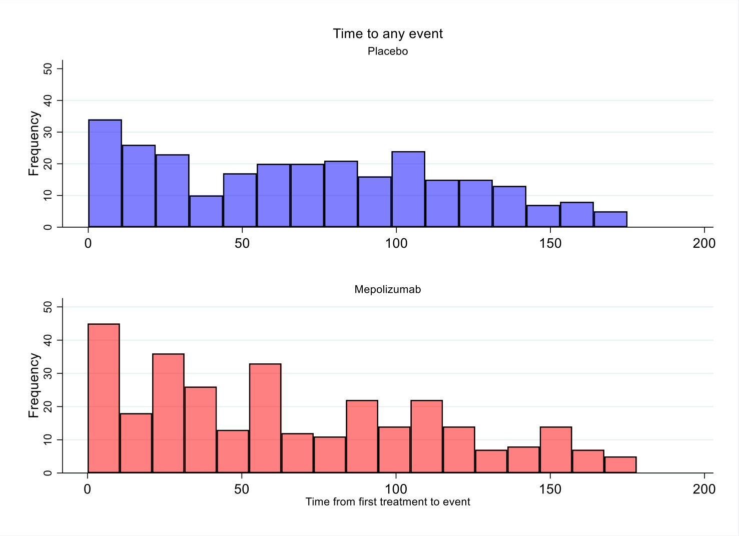

Figure description: Histogram of time of events (rather than categorising into arbitrary time periods). Not just looking at time-to-first event or maximum event, includes time of every event.

Figure A.21: Nelson-Aalen cumulative hazards

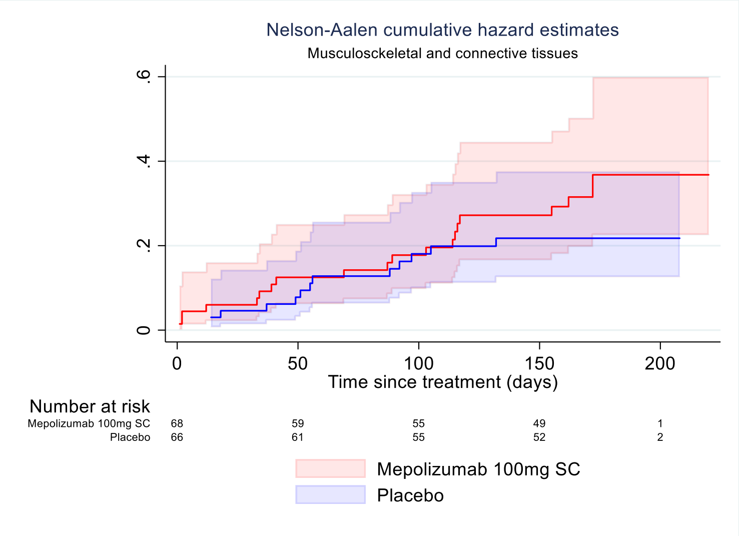

Figure description: Cumulative hazards by treatment arm with 95% confidence intervals and a table of numbers at risk

Adaptions considered: Without 95% CI &/or risk table.

Supplement 3 - Visualisations considered but not recommended

Figure A.22: Mean cumulative duration

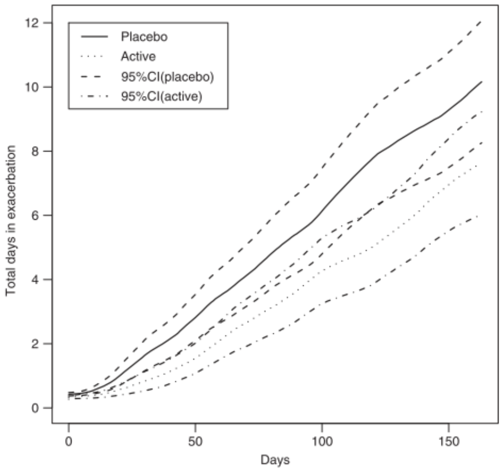

Figure 1 Mean duration of exacerbation with 95% CI based on the robust variance estimate for recurrent pulmonary exacerbations in patients with fibrosis treated with placebo and rhDNase (Fuchs et al., 1994).

Figure description: Displays the mean cumulative duration (MCD) as a function of time by treatment arm. The MCD is a non-parametric estimate of the mean cumulative duration of events per participant. Accounts for repeated occurrence of an event in a participant. Includes 95% confidence interval bands across follow-up. *Reprinted from: Wang, J. and G. Quartey (2012). "Nonparametric estimation for cumulative duration of adverse events." Biometrical Journal **54**(1): 61-74 with permission from John Wiley & Sons.*

Adaptions considered: Different colours for each treatment group

Figure A.23: Bar chart of median time to event

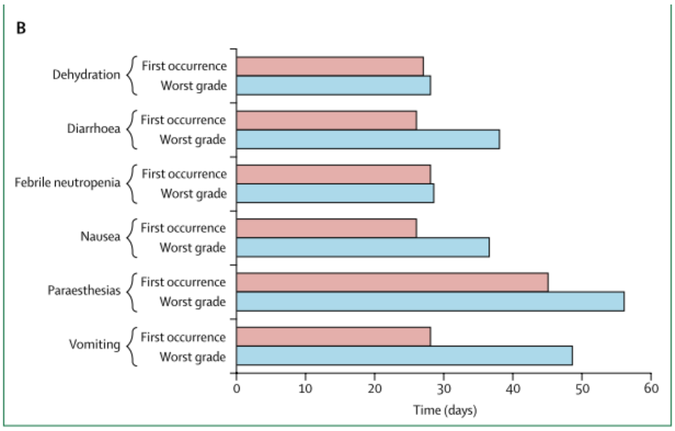

***Figure 3*: Time-to-event analyses for adverse events using the Toxicity over Time package**
(A) Time to grade 2 or worse diarrhoea in patients given FOLFOX and IROX in NCCTG N9741.[36] (B) Median time to first occurrence and worst grade toxic effects in patients given IROX in NCCTG N9741.[36] The figures capture the time profile of adverse events from these regimens. IROX=irinotecan and oxaliplatin. FOLFOX=leucovorin, fluorouracil, and oxaliplatin.

Figure description: Horizontal bar graph of median time to first (and worst grade) event. Height/length of each bar represents the median time to event. Different events are displayed along the y-axis. Time is displayed on the x-axis. Use separate bars for each treatment arm instead of first and worst event. **Caution:** This is taken from a publication in the Lancet Oncology but we think it could be very misleading since: it doesn't account for censoring or show how the denominator changes over time; and it doesn't include any information on the number of participants that have these events. *Reprinted from: Thanarajasingam G, Atherton PJ, Novotny PJ, Loprinzi CL, Sloan JA, Grothey A. Longitudinal adverse event assessment in oncology clinical trials: the Toxicity over Time (ToxT) analysis of Alliance trials NCCTG N9741 and 979254. Lancet Oncol. 2016;17(5):663-670. doi:10.1016/S1470-2045(16)00038-3 with permission from Elsevier.*

Adaptions considered: Include separate bars for each treatment group instead of first and worst event

Supplement 3 - Visualisations considered but not recommended

Figure A.24: Empirical distribution of maximum change

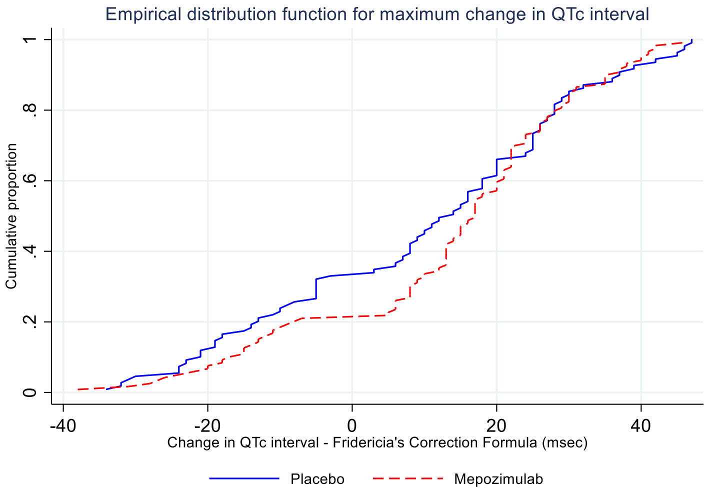

Figure description: Displays the cumulative proportion of participants on the y-axis with a change in QTc less than or equal to the corresponding value on the x-axis. Displays maximum change for each participant. Treatment arms displayed in different colours. *Original plot first proposed in: Amit, O., et al. (2008). "Graphical approaches to the analysis of safety data from clinical trials." <u>Pharmaceutical Statistics</u>* ***7****(1): 20-35.*

Figure A.25: Box plot of change values

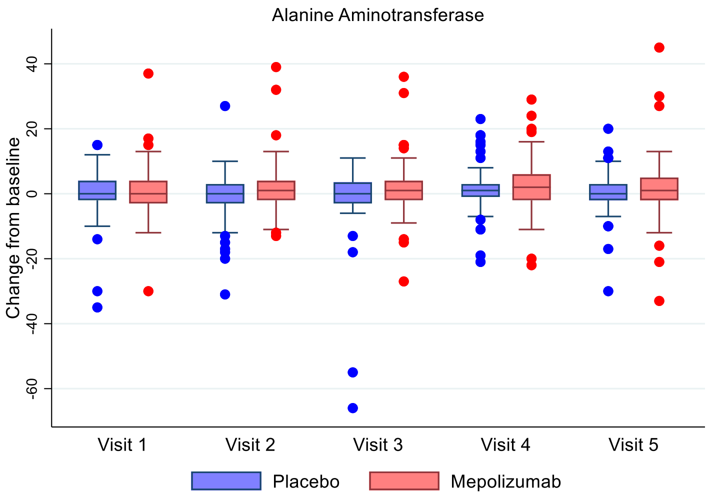

Figure description: Box plot of change from baseline across visits by treatment arm. Treatment arms displayed in different colours.

Supplement 3 - Visualisations considered but not recommended

Figure A.26: Delta plot

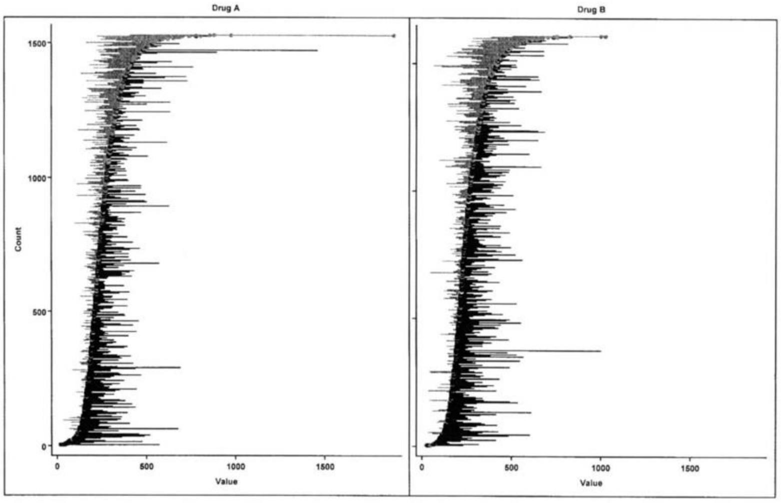

FIGURE 2a. Side-by-side Delta plots of baseline and last follow-up platelet values.

Figure description: Displays individual participant changes. The ends of each line indicate baseline and last follow-up values read from the x-axis for individual participants. Arranged according to baseline values. Y-axis tracks cumulative number of lines/participants. **Caution**: We do not find this plot very intuitive/helpful but included for comprehension. *Reprinted from: Chuang-Stein, C., et al. (2001). "Recent Advancements in the Analysis and Presentation of Safety Data."* *Drug Information Journal* *35(2): 377-397 under the terms of the Creative Commons CC BY License.*

Figure A.27: E-dish plot

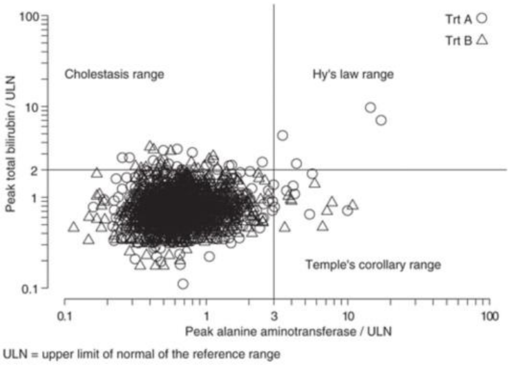

Figure 2 e-DISH-like plot. Notes: Plot of peak bilirubin (/ULN) *vs* peak ALT (/ULN). This figure shows peak values for total bilirubin and aminotransferases by treatment groups. Significant elevations of aminotransferases ('Temple's Corollary range') and, especially, abnormalities in the 'Hy's Law Range' should be carefully analyzed as potential signals for drug-induced liver injury [11]. Please see Refs [12] and [13] for further information on the use of this plot

Figure description: Specific scatterplot for maximum ALT, AST & Bilirubin values. Plots peak bilirubin vs peak ALT or AST. **Note**: Again, we need to consider where, if anywhere, we would advise using such an image. Perhaps better suited to monitoring of ongoing trials. *Reprinted from: Xia HA, Crowe BJ, Schriver RC, Oster M, Hall DB. Planning and core analyses for periodic aggregate safety data reviews. Clin Trials. 2011;8(2):175-182. doi:10.1177/1740774510395635 with permission from Sage Publishing*

Supplement 3 - Visualisations considered but not recommended

Figure A.28: Vector plot

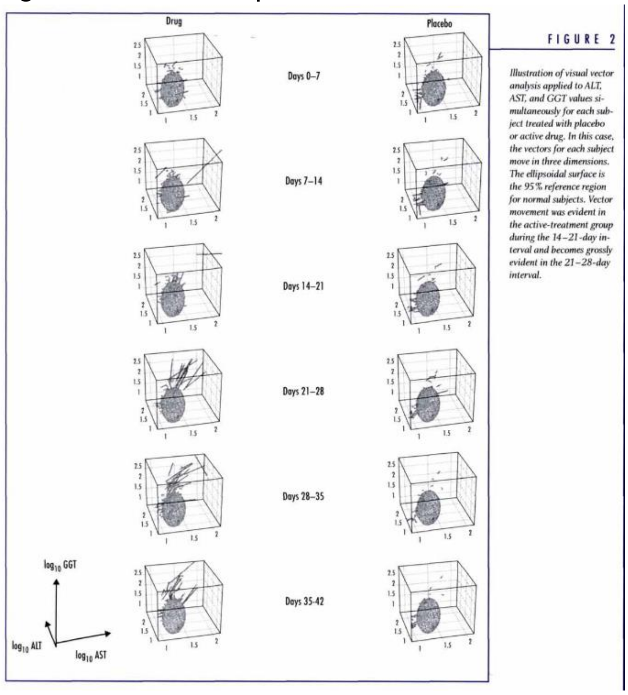

Figure description: Simultaneously displays individual participant changes across three laboratory values. Grey circle indicates the 95% reference range of values for ‘normal’ subjects. **Caution**: 3D images may hide some information when viewed in a static format so we do not explore this image any further. *Reprinted from: Trost, D. C. and J. W. Freston (2008). "Vector Analysis to Detect Hepatotoxicity Signals in Drug Development." Therapeutic Innovation & Regulatory Science 42(1): 27-34 under the terms of the Creative Commons CC BY License.*
