# Supplementary material for: Visualising harms in publications of randomised controlled trials: consensus and recommendations
Source: BMJ. 2022 May 16;377:e068983. doi: 10.1136/bmj-2021-068983 (PMC9108928; doi:10.1136/bmj-2021-068983)
Supplement: Supplementary file 4 — Web appendix: Supplement 4: Example R code and dataset to create the dot plot [file phir068983.ww4.pdf]

Supplement 4: Example R code and dataset to create the dot plot

Example R code

```
#####################################################################################

# R Code for Dot plot to visualize AE and harm profiles in two-arm randomised controlled trials

# Graham Wheeler and Rachel Phillips, Imperial Clinical Trials Unit, Imperial College London

# Based on code developed by Riaz Qureshi, Johns Hopkins University available
https://github.com/rquresh/HarmsVisualization/blob/main/DotPlot.Rmd

#####################################################################################


### Set Working directory to be whichever folder contains the data

setwd("")

### Import the comma separate value (.csv) file

SummaryDataset <- read.csv("example_dataset_dotplot_summary_level.csv", sep=",", fileEncoding = 'UTF-8-BOM')

RDSortedBS <- SummaryDataset[order(-SummaryDataset$relrisk ),]


### Create subset of full data with the elements that we need for each half of the plot

BSRisk <- subset(RDSortedBS, select=c(event,Intervention,Placebo,relrisk))

BSRisk$event <- factor(BSRisk$event, levels = BSRisk$event[order(-BSRisk$relrisk)])

BSRiskRatio<- subset(RDSortedBS, select=c(event,relrisk,lowerCIRR,upperCIRR))

BSRiskRatio$event <- factor(BSRiskRatio$event, levels = BSRiskRatio$event[order(-BSRiskRatio$relrisk)])


### If any of the packages below have not yet been installed, use "install.packages("<package>") as below:

# install.packages("reshape")

# install.packages("ggplot2")

# install.packages("scales")


library(reshape)

ByGroup <- melt(BSRisk, id=c("event"))

ByGroup <- ByGroup[ByGroup$variable != "relrisk",]


library(ggplot2)


### "left" is a ggplot object of the group-specific risks (percentage of participants experiencing each type of event)

left <- ggplot(ByGroup, aes(x=value, y=event, fill=variable)) +

  geom_dotplot(binaxis='y', stackdir='center', dotsize = 0.5) +

  scale_fill_manual(values=c("red", "blue")) +
```

Supplement 4: Example R code and dataset to create the dot plot

```
  ggtitle("") +
  ylab("Body system") +
  scale_x_continuous(name = "Percentage of participants (%)",  ) +
  scale_y_discrete(limits = rev(levels(ByGroup$event))) +
  theme(legend.position="bottom",
        legend.title = element_blank(),
        panel.background = element_blank(),
        panel.border = element_blank(),
        panel.grid.major.y = element_line(color = "grey", size = 0.1, linetype = 1),
        axis.ticks.x = element_line(colour = "black"),
        axis.line = element_line(color = 'black'))

### You can plot "left" on its own to check its appearance:
# left

### "right" is a ggplot object of the effect estimate and corresponding confidence interval
library(scales)
right <-  ggplot(BSRiskRatio, aes(y=event, x=relrisk, xmin=lowerCIRR, xmax=upperCIRR, fill = "Relative risk with 95% CI")) +
  ggstance::geom_pointrangeh(aes(xmin = lowerCIRR, xmax = upperCIRR)) +
  ggtitle("")+
  geom_vline(xintercept = 1, linetype = 2, colour = "blue", size = 0.75) +
  scale_x_continuous(name = "Relative risk with 95% CI",
                     trans = log2_trans(),
                     breaks = c(0.10,0.5,1,2, 5, 10, 50,100,220),
                     labels = as.character(c(0.10,0.5,1,2, 5, 10, 50,100,220))) +
  scale_y_discrete(limits = rev(levels(BSRiskRatio$event))) +
  theme(legend.position="bottom",
        legend.title = element_blank(),
        axis.title.y = element_blank(),
        axis.text.y = element_blank(),
        panel.background = element_blank(),
        panel.border = element_blank(),
        panel.grid.major.y = element_line(color = "grey", size = 0.1, linetype = 1),
        axis.ticks.y = element_blank(),
        axis.ticks.x = element_line(colour = "black"),
```

Supplement 4: Example R code and dataset to create the dot plot

```
        axis.text.x = element_text(angle=0, hjust=0.5, size=10),
        axis.line.x = element_line(size = 0.5, linetype = "solid", colour = "black"))


### You can plot "right" on its own to check its appearance:
# right


### This code creates the table format for the number of patients experiencing an event per arm,
### or the total number of events of each type per arm


tab_base <- ggplot(RDSortedBS, aes(y=event)) +
  ylab(NULL) + xlab(" ") +   scale_y_discrete(limits = rev(levels(BSRiskRatio$event))) +
  theme(plot.title = element_text(hjust = 0.5, size=12), ## centering title on text
        axis.text.x=element_text(color="white"), ## need text to be printed so it stays aligned with figure but white so it's invisible
        axis.line=element_blank(),
        axis.text.y=element_blank(),axis.ticks=element_blank(),
        axis.title.y=element_blank(),legend.position="bottom", legend.title = element_blank(),
        panel.background=element_blank(),panel.border=element_blank(),panel.grid.major=element_blank(),
        panel.grid.minor=element_blank(),plot.background=element_blank())


### Tables of number of participants experiencing each harm type for Intervention (I) and Placebo (P) arms
t_I_n<-tab_base + geom_text(aes(x=1, label = eventn1, hjust = "middle")) + ggtitle(expression("I"["n"]))
t_P_n<-tab_base + geom_text(aes(x=1, label = eventn2, hjust = "middle")) + ggtitle(expression("P"["n"]))


### Tables of total number of events per harm type for Intervention (I) and Placebo (P) arms
t_I_event<-tab_base + geom_text(aes(x=1, label = n_events1, hjust = "middle")) + ggtitle(expression("I"["event"]))
t_P_event<-tab_base + geom_text(aes(x=1, label = n_events2, hjust = "middle")) + ggtitle(expression("P"["event"]))



### Now put "left", "right", and tables of your choice together in one figure.
### Again, install any of the packages below if they have not yet been installed:
# install.packages("ggpubr")
# install.packages("cowplot")
library(ggpubr)
library(cowplot)
```

Supplement 4: Example R code and dataset to create the dot plot

```
### Dot plot Version 1 - only number of participants with each events

DotPlot <- plot_grid(left, right, t_I_n, t_P_n, nrow = 1, align = "h", rel_widths = c(3,2,1,1), axis = "b")

### NB: may take a few seconds to generate

annotate_figure(DotPlot,

        bottom = text_grob(bquote("I:Intervention (N = "*.(RDSortedBS[1,"N1"])*"), P:Placebo (N = "*.(RDSortedBS[1,"N2"])*"); X"["n"]*"= number of participants in arm X with AE"),

                color = "black", face = "bold", size = 10),

        top = text_grob("", color = "black", face = "bold", size = 10))


### Dot plot Version 2 - number of participants with each event and total number of events

DotPlot <- plot_grid(left, right, t_I_n, t_I_event, t_P_n, t_P_event, nrow = 1, align = "h", rel_widths = c(3,2,0.5,0.5,0.5,0.5), axis = "b")

### NB: may take a few seconds to generate

annotate_figure(DotPlot,

        bottom = text_grob(bquote("I:Intervention (N = "*.(RDSortedBS[1,"N1"])*"), P:Placebo (N = "*.(RDSortedBS[1,"N2"])*"); X"["n"]*"= number of participants in arm X with AE, X"["event"]*"= number of AEs in arm X"),

                color = "black", face = "bold", size = 10),

        top = text_grob("", color = "black", face = "bold", size = 10))


#######

# END #

#######
```

Supplement 4: Example R code and dataset to create the dot plot

Example dataset

r1	eventn1	N1	r2	eventn2	N2	Intervention	Placebo	risk_diff	seRD	lowerRD	upperRD	n_events	n_events1	n_events2	p_val	log_p_val	event	relrisk	logRR	stderrRR	loglowerCIRR	logupperCIRR	lowerCIRR	upperCIRR
0.766667	23	30	1	31	31	76.66666	100	-0.23333	0.07722	-0.38468	-0.08198	158	68	90	0.004666	2.331021	Blood and lymphatic	0.766667	-0.2657	0.100722	-0.46312	-0.06829	0.629318	0.933991
0.233333	7	30	0.258065	8	31	23.33333	25.80645	-0.02473	0.110179	-0.24068	0.191219	23	14	9	1	2.18E-14	Dermatological	0.904167	-0.10074	0.44974	-0.98223	0.780748	0.374475	2.183105
0.1	3	30	0.096774	3	31	10	9.67742	0.003226	0.076287	-0.1463	0.152748	6	3	3	1	-4.82E-16	Eyes, ear, nose, thr	1.033333	0.03279	0.77529	-1.48678	1.552359	0.2261	4.722598
0.533333	16	30	0.83871	26	31	53.33333	83.87096	-0.30538	0.112517	-0.52591	-0.08484	94	29	65	0.013428	1.871977	Gastrointestinal	0.635897	-0.45272	0.18807	-0.82133	-0.0841	0.439844	0.919338
0.166667	5	30	0.032258	1	31	16.66667	3.225807	0.134409	0.075078	-0.01274	0.281561	8	7	1	0.103516	0.984994	Infection	5.166667	1.642228	1.065086	-0.44534	3.729797	0.640606	41.67064
0.233333	7	30	0.193548	6	31	23.33333	19.35484	0.039785	0.104872	-0.16576	0.245333	29	14	15	0.762211	0.117925	Neurological	1.205556	0.186941	0.493895	-0.78109	1.154975	0.457905	3.173944
0.4	12	30	0.225806	7	31	40	22.58065	0.174194	0.116787	-0.05471	0.403097	23	13	10	0.173665	0.760288	Other	1.771429	0.571786	0.400748	-0.21368	1.357253	0.807607	3.885504
0.133333	4	30	0.096774	3	31	13.33333	9.67742	0.036559	0.081679	-0.12353	0.19665	14	10	4	0.707182	0.150469	Psychiatric	1.377778	0.320472	0.719543	-1.08983	1.730776	0.336273	5.645032
0.033333	1	30	0.129032	4	31	3.333333	12.90323	-0.0957	0.068552	-0.23006	0.038662	9	3	6	0.353987	0.451012	Renal and urinary	0.258333	-1.3535	1.088305	-3.48658	0.779574	0.030605	2.180543
0.033333	1	30	0.129032	4	31	3.333333	12.90323	-0.0957	0.068552	-0.23006	0.038662	6	1	5	0.353987	0.451012	Respiratory	0.258333	-1.3535	1.088305	-3.48658	0.779574	0.030605	2.180543
