# Supplementary material for: Visualising harms in publications of randomised controlled trials: consensus and recommendations
Source: BMJ. 2022 May 16;377:e068983. doi: 10.1136/bmj-2021-068983 (PMC9108928; doi:10.1136/bmj-2021-068983)
Supplement: Supplementary file 5 — Web appendix: Supplement 5: Table and figures summarising initial appraisals of all plots by outcome type [file phir068983.ww5.pdf]

Supplement 5: Table and figures summarising initial appraisals of all plots by outcome type

Table A.2a: Plots suitable for **Multiple Binary Outcomes –** summary of scores

**Appraisal criteria**	**Volcano**		**Alternative volcano 1**		**Alternative volcano 2**		**Alternative volcano 3**		**Dot plot**		**Bar**	
	n	Mean (SD)	n	Mean (SD)	n	Mean (SD)	n	Mean (SD)	n	Mean (SD)	n	Mean (SD)
		Median (Min, Max)		Median (Min, Max)		Median (Min, Max)		Median (Min, Max)		Median (Min, Max)		Median (Min, Max)
1.Effect size	21	4.0 (1.0)	21	4.0 (0.8)	21	2.3 (1.2)	15	3.2 (1.3)	21	4.3 (1.1)	21	2.8 (1.1)
		4 (1, 5)		4 (3, 5)		2 (1, 5)		4 (1, 5)		5 (1, 5)		3 (1, 5)
2.Direction of effect	21	4.1 (1.1)	21	4.0 (1.2)	20	2.9 (1.4)	14	2.8 (1.7)	21	4.5 (0.8)	21	4.0 (1.1)
		4 (1, 5)		4 (1, 5)		3 (1, 5)		3 (1, 5)		5 (2, 5)		4 (1, 5)
3.Uncertainty	21	1.8 (1.1)	21	1.8 (0.7)	21	2.0 (1.0)	14	1.3 (0.5)	21	4.4 (1.0)	21	1.3 (0.6)
		1 (1, 5)		2 (1, 3)		2 (1, 5)		1 (1, 2)		5 (1, 5)		1 (1, 3)
4.Supplementary data needed	21	2.1 (1.1)	21	2.5 (1.1)	21	2.0 (1.1)	13	2.2 (1.1)	21	3.8 (1.2)	21	2.6 (1.1)
		2 (1, 4)		2 (1, 5)		2 (1, 5)		2 (1, 4)		4 (1, 5)		2 (1, 5)
5.Understandable	21	2.9 (0.8)	21	3.0 (1.1)	21	2.3 (1.0)	14	1.5 (0.7)	21	4.2 (0.8)	21	4.8 (0.4)
		3 (2, 4)		3 (1, 5)		2 (1, 4)		1 (1, 3)		4 (2, 5)		5 (4, 5)
6.Understandable non-stats	21	2.3 (0.9)	21	2.9 (1.0)	21	2.3 (0.9)	14	1.4 (0.6)	21	4.0 (0.9)	20	4.7 (0.6)
		2 (1, 4)		3 (1, 4)		2 (1, 4)		1 (1, 3)		4 (2, 5)		5 (3, 5)
7.Multi-arm studies	21	2.0 (0.7)	21	2.1 (0.9)	21	3.8 (1.0)	14	1.9 (1.0)	21	3.0 (1.0)	21	4.7 (0.7)
		2 (1, 3)		2 (1, 4)		4 (1, 5)		2 (1, 4)		3 (1, 5)		5 (2, 5)
8.Limits numbers	21	3.2 (0.8)	21	3.9 (0.8)	21	3.7 (1.1)	14	3.1 (1.2)	21	3.7 (0.8)	21	3.9 (0.7)
		3 (2, 5)		4 (2, 5)		4 (1, 5)		3 (1, 5)		4 (2, 5)		4 (3, 5)
**9.Overall score***	**21**	**19.2 (4.5)**	**21**	**20.3 (4.0)**	**21**	**17.3 (5.0)**	**21**	**9.6 (7.6)**	**21**	**28.1 (5.0)**	**21**	**24.6 (3.2)**
		**19 (11, 27)**		**20 (12, 28)**		**17 (7, 28)**		**11 (0, 23)**		**29 (15, 34)**		**26 (18, 30)**
10. Suitable for publication	21	3.3 (1.1)	21	3.6 (1.0)	19	2.5 (1.4)	14	1.7 (1.1)	20	4.3 (0.9)	20	3.8 (1.0)
		3 (1, 5)		4 (1, 5)		2 (1, 5)		1 (1, 4)		5 (2, 5)		4 (2, 5)
11. Suitable for final report	20	3.5 (1.1)	20	3.7 (1.0)	19	2.5 (1.4)	14	1.8 (1.3)	20	4.3 (0.7)	20	4.0 (0.9)
		4 (1, 5)		4 (1, 5)		2 (1, 5)		1 (1, 5)		5 (3, 5)		4 (2, 5)
12. Suitable for interim analysis	20	3.2 (1.2)	20	3.5 (1.1)	19	2.4 (1.3)	14	1.5 (0.9)	20	4.3 (0.7)	20	4.1 (0.7)
		3 (1, 5)		4 (1, 5)		2 (1, 5)		1 (1, 4)		4 (3, 5)		4 (3, 5)
13.Exploratory analysis	20	3.7 (0.6)	20	3.4 (0.8)	19	2.8 (0.9)	14	1.9 (1.1)	19	3.8 (0.8)	19	3.7 (1.1)
		4 (3, 5)		4 (2, 5)		3 (1, 4)		2 (1, 4)		4 (2, 5)		4 (1, 5)
14.Explanatory analysis	20	3.1 (0.7)	20	3.3 (0.9)	19	2.5 (1.0)	14	1.9 (1.1)	19	4.1 (0.8)	19	3.5 (0.9)
		3 (2, 4)		3 (2, 5)		3 (1, 4)		2 (1, 4)		4 (3, 5)		3 (2, 5)
**Ranking**	**18**	**5.6 (2.1)**	**18**	**4.8 (1.8)**	**18**	**6.6 (2.9)**	**14**	**9.8 (2.3)**	**20**	**1.6 (1.6)**	**17**	**3.8 (1.9)**
		**5 (2, 10)**		**5 (2, 9)**		**7 (1, 12)**		**11 (4, 12)**		**1 (1, 7)**		**4 (1, 9)**

* Overall score is the sum total of questions 1-7

Supplement 5: Table and figures summarising initial appraisals of all plots by outcome type

Table A.2b: Plots suitable for **Multiple Binary Outcomes –** summary of scores

Question	Tendril		Heat map		Stacked bar chart		Stacked bar chart - counts		Star		Alluvial	
	n	Mean (SD) / Median (Min, Max)	n	Mean (SD) / Median (Min, Max)	n	Mean (SD) / Median (Min, Max)	n	Mean (SD) / Median (Min, Max)	n	Mean (SD) / Median (Min, Max)	n	Mean (SD) / Median (Min, Max)
1.Effect size	21	1.3 (0.6)	20	3.0 (0.7)	21	3.1 (1.2)	12	2.4 (1.1)	21	2.0 (0.9)	21	1.4 (0.6)
		1 (1, 3)		3 (2, 4)		3 (1, 5)		3 (1, 4)		2 (1, 4)		1 (1, 3)
2.Direction of effect	21	1.6 (1.0)	20	3.6 (1.0)	21	4.1 (0.9)	12	3.3 (1.2)	21	2.7 (1.0)	21	1.3 (0.6)
		1 (1, 5)		4 (1, 5)		4 (1, 5)		4 (1, 5)		3 (1, 4)		1 (1, 3)
3.Uncertainty	21	1.2 (0.5)	20	1.3 (0.7)	21	1.4 (0.6)	12	1.3 (0.5)	20	1.1 (0.4)	20	1.1 (0.4)
		1 (1, 3)		1 (1, 4)		1 (1, 3)		1 (1, 2)		1 (1, 2)		1 (1, 2)
4.Supplementary data needed	21	1.6 (1.2)	20	2.1 (0.8)	21	3.2 (0.9)	12	3.0 (1.0)	20	1.9 (1.1)	20	1.9 (1.4)
		1 (1, 5)		2 (1, 4)		3 (2, 5)		3 (2, 5)		2 (1, 5)		1 (1, 5)
5.Understandable	21	1.2 (0.5)	20	3.1 (1.0)	21	4.7 (0.5)	12	4.4 (0.8)	20	2.3 (0.9)	20	2.1 (1.0)
		1 (1, 3)		3 (2, 5)		5 (4, 5)		5 (3, 5)		2 (1, 4)		2 (1, 4)
6.Understandable non-stats	21	1.0 (0.2)	20	2.9 (1.0)	21	4.5 (0.6)	12	4.1 (1.0)	20	1.9 (0.7)	20	1.9 (0.9)
		1 (1, 2)		3 (2, 5)		5 (3, 5)		4 (2, 5)		2 (1, 3)		2 (1, 4)
7.Multi-arm studies	21	1.4 (0.7)	20	1.9 (0.9)	21	4.1 (1.2)	12	4.3 (1.2)	20	3.9 (1.4)	20	1.6 (0.7)
		1 (1, 3)		2 (1, 4)		5 (1, 5)		5 (2, 5)		4 (1, 5)		1 (1, 3)
8.Limits numbers	21	3.3 (1.2)	19	3.6 (1.0)	21	3.5 (0.7)	11	3.5 (0.7)	19	2.9 (1.0)	20	2.5 (1.1)
		4 (1, 5)		4 (1, 5)		3 (2, 5)		3 (3, 5)		3 (1, 5)		3 (1, 4)
**9.Overall score***	**21**	**9.3 (2.4)**	**21**	**17.1 (5.1)**	**21**	**25.1 (2.8)**	**21**	**13.0 (12.0)**	**21**	**15.4 (5.0)**	**21**	**10.9 (3.5)**
		**9 (7, 14)**		**17 (0, 24)**		**25 (19, 29)**		**15 (0, 28)**		**16 (2, 23)**		**11 (2, 21)**
10. Suitable for publication	20	1.5 (0.9)	20	2.8 (0.9)	21	4.0 (0.9)	11	3.5 (1.1)	20	2.2 (1.2)	19	1.7 (1.1)
		1 (1, 4)		3 (1, 4)		4 (2, 5)		4 (2, 5)		2 (1, 5)		1 (1, 4)
11. Suitable for final report	20	1.8 (1.1)	20	2.7 (0.9)	21	4.0 (0.8)	11	3.6 (1.0)	20	2.4 (1.3)	19	2.0 (1.1)
		1 (1, 4)		3 (1, 4)		4 (2, 5)		4 (2, 5)		2 (1, 5)		2 (1, 4)
12. Suitable for interim analysis	20	2.1 (1.4)	20	2.5 (0.9)	21	4.1 (0.8)	11	3.6 (1.0)	20	2.3 (1.2)	19	2.1 (1.2)
		2 (1, 5)		3 (1, 4)		4 (2, 5)		4 (2, 5)		2 (1, 5)		2 (1, 4)
13.Exploratory analysis	20	3.0 (1.2)	19	3.1 (1.0)	20	4.0 (0.9)	11	3.5 (1.1)	19	2.9 (1.1)	19	2.8 (1.5)
		3 (1, 5)		3 (1, 4)		4 (2, 5)		4 (2, 5)		3 (1, 5)		3 (1, 5)
14.Explanatory analysis	19	1.7 (1.0)	19	2.6 (1.0)	20	4.0 (1.1)	11	3.3 (1.3)	19	2.2 (1.1)	18	1.7 (0.8)
		1 (1, 5)		3 (1, 5)		4 (1, 5)		3 (1, 5)		2 (1, 5)		2 (1, 3)
**Ranking**	**18**	**10.2 (2.3)**	**17**	**7.5 (1.8)**	**19**	**2.4 (1.1)**	**11**	**4.9 (2.3)**	**17**	**8.0 (2.3)**	**17**	**9.9 (2.6)**
		**11 (2, 12)**		**8 (3, 10)**		**2 (1, 5)**		**4 (2, 10)**		**8 (3, 12)**		**11 (3, 12)**

* Overall score is the sum total of questions 1-7

Supplement 5: Table and figures summarising initial appraisals of all plots by outcome type

Figure A.29: Multiple binary outcomes

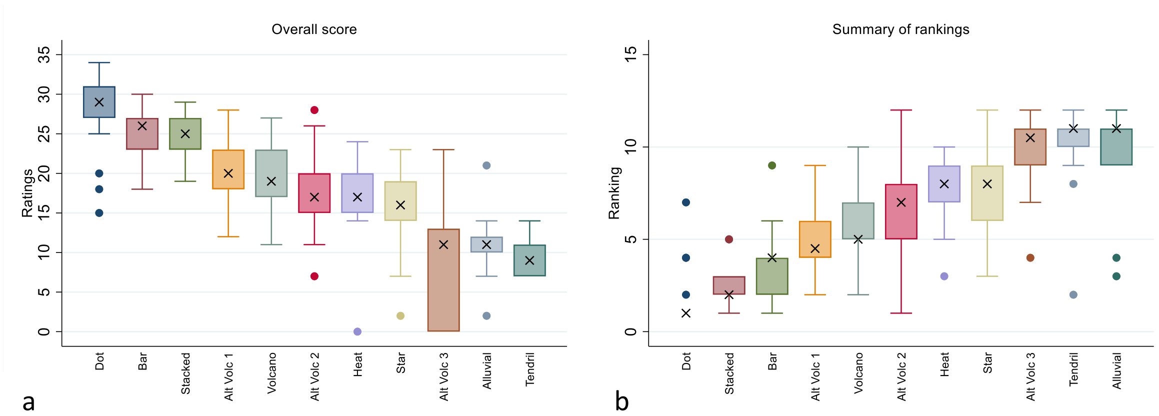

a. Box plot of overall scores ordered by highest to lowest mean values (higher scores indicate better performance). **b. Box plot of rankings** ordered by best to worst mean rank (lower ranking indicates preferred plot).

Note: X indicates median values. Excludes summary for stacked bar chart of counts as only limited numbers scored this plot

Supplement 5: Table and figures summarising initial appraisals of all plots by outcome type

Table A.3: Plots suitable for **Single Binary Outcomes –** summary of scores

Question	Bar chart	
	n	Mean (SD)<br>Median (Min, Max)
1.Effect size	23	2.1 (0.9)
		2 (1, 4)
2.Direction of effect	23	2.3 (1.0)
		2 (1, 4)
3.Uncertainty	23	1.1 (0.3)
		1 (1, 2)
4.Supplementary data needed	23	2.5 (1.2)
		2 (1, 4)
5.Understandable	23	4.0 (1.2)
		4 (1, 5)
6.Understandable non-stats	23	3.9 (0.9)
		4 (2, 5)
7.Multi-arm studies	23	3.9 (0.9)
		4 (2, 5)
8.Limits numbers	23	3.2 (1.2)
		3 (1, 5)
**9.Overall score**	**23**	**19.8 (3.3)**
		**20 (11, 26)**
10. Suitable for publication	22	3.1 (0.9)
		3 (1, 4)
11. Suitable for final report	22	3.4 (1.0)
		4 (1, 4)
12. Suitable for interim analysis	22	3.5 (1.0)
		4 (1, 5)
13.Exploratory analysis	22	3.1 (1.0)
		3 (1, 5)
14.Explanatory analysis	22	3.0 (1.0)
		3 (1, 5)
**Ranking**	**6**	**1.0 (0.0)**
		**1 (1, 1)**

* Overall score is the sum total of questions 1-7

Supplement 5: Table and figures summarising initial appraisals of all plots by outcome type

Figure A.30: Single binary outcomes

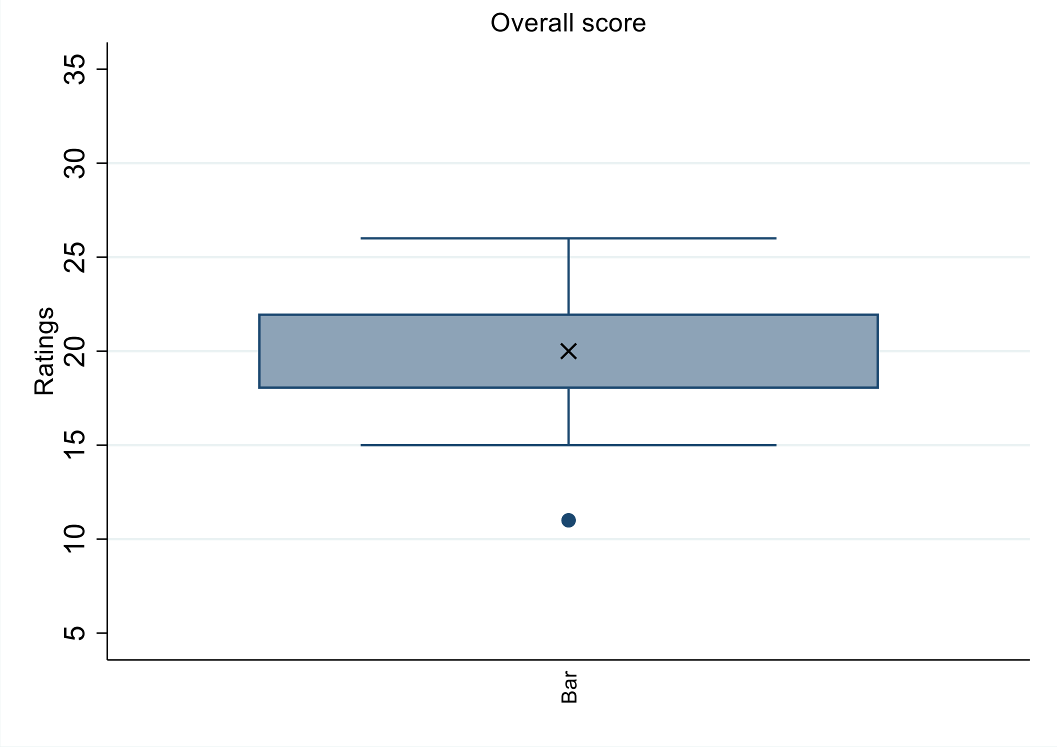

Box plot of overall scores, ordered by highest to lowest mean values (higher scores indicate better performance).

Note: X indicates median values.

Supplement 5: Table and figures summarising initial appraisals of all plots by outcome type

Table A.4: Plots suitable for **Multiple Time-to-Event Outcomes –** summary of scores

Question	Matrix of cumulative hazards		Bar chart		Alternative bar chart		Alternative survival plot 1		Alternative survival plot 2	
	n	Mean (SD) / Median (Min, Max)	n	Mean (SD) / Median (Min, Max)	n	Mean (SD) / Median (Min, Max)	n	Mean (SD) / Median (Min, Max)	n	Mean (SD) / Median (Min, Max)
1.Effect size	21	3.4 (1.0)	21	2.4 (1.2)	21	2.3 (1.3)	21	2.3 (0.8)	18	1.6 (0.8)
		4 (2, 5)		2 (1, 4)		2 (1, 4)		2 (1, 4)		1 (1, 4)
2.Direction of effect	21	3.9 (0.8)	21	3.0 (1.3)	21	2.8 (1.1)	21	2.9 (1.2)	18	1.4 (0.6)
		4 (2, 5)		3 (1, 5)		3 (1, 4)		3 (1, 5)		1 (1, 3)
3.Uncertainty	21	2.4 (1.5)	21	1.4 (0.8)	21	1.5 (0.8)	21	1.1 (0.3)	18	1.2 (0.4)
		2 (1, 5)		1 (1, 4)		1 (1, 3)		1 (1, 2)		1 (1, 2)
4.Supplementary data needed	20	2.5 (1.1)	21	2.1 (1.1)	21	1.9 (0.8)	21	2.0 (1.1)	18	1.7 (1.1
		2 (1, 5)		2 (1, 4)		2 (1, 3)		2 (1, 5)		1 (1, 5)
5.Understandable	21	4.1 (0.7)	21	3.2 (1.1)	21	2.7 (1.4)	21	2.2 (1.0)	18	2.5 (1.2)
		4 (3, 5)		3 (1, 5)		3 (1, 5)		2 (1, 5)		3 (1, 4)
6.Understandable non-stats	20	3.2 (1.0)	21	3.0 (1.1)	21	2.3 (1.2)	21	2.0 (1.0)	18	2.1 (1.2)
		3 (1, 5)		3 (1, 5)		2 (1, 5)		2 (1, 5)		2 (1, 4)
7.Multi-arm studies	21	4.5 (0.6)	21	3.6 (1.3)	21	3.7 (1.4)	21	1.9 (0.9)	18	2.4 (1.2)
		5 (3, 5)		4 (1, 5)		4 (1, 5)		2 (1, 4)		3 (1, 5)
8.Limits numbers	21	2.3 (1.1)	21	3.0 (1.0)	19	3.8 (1.3)	21	3.2 (1.1)	18	2.3 (1.0)
		2 (1, 5)		3 (1, 4)		4 (1, 5)		3 (1, 5)		3 (1, 4)
**9.Overall score**	**21**	**24.0 (4.8)**	**21**	**19.0 (5.4)**	**21**	**17.5 (6.6)**	**21**	**14.6 (4.2)**	**21**	**11.2 (6.1)**
		**23 (18, 35)**		**20 (7, 28)**		**20 (7, 29)**		**15 (7, 22)**		**13 (0, 20)**
10. Suitable for publication	20	3.4 (1.1)	20	2.2 (1.1)	20	2.2 (1.4)	20	1.8 (0.9)	18	1.9 (1.0)
		4 (1, 5)		2 (1, 4)		2 (1, 5)		2 (1, 4)		2 (1, 4)
11. Suitable for final report	20	3.8 (1.0)	20	2.3 (1.3)	20	2.3 (1.3)	20	1.8 (0.9)	18	1.9 (1.0)
		4 (1, 5)		2 (1, 4)		2 (1, 5)		2 (1, 4)		2 (1, 4)
12. Suitable for interim analysis	20	3.8 (1.0)	20	2.3 (1.4)	20	2.3 (1.3)	20	2.2 (1.4)	18	2.1 (1.0)
		4 (1, 5)		2 (1, 5)		2 (1, 5)		2 (1, 5)		2 (1, 4)
13.Exploratory analysis	20	3.7 (1.1)	20	2.5 (1.3)	20	2.6 (1.3)	20	3.1 (1.3)	17	2.4 (1.4)
		4 (1, 5)		3 (1, 4)		3 (1, 5)		3 (1, 5)		2 (1, 5)
14.Explanatory analysis	20	3.5 (1.1)	20	2.2 (1.3)	20	2.4 (1.2)	20	2.4 (1.0)	17	2.2 (1.1)
		4 (1, 5)		2 (1, 4)		3 (1, 4)		2 (1, 4)		2 (1, 4)
**Ranking**	**18**	**1.3 (0.6)**	**16**	**3.4 (1.3)**	**17**	**3.5 (1.3)**	**16**	**3.1 (1.1)**	**15**	**4.5 (0.7)**
		**1 (1, 3)**		**4 (1, 5)**		**3 (1, 5)**		**3 (2, 5)**		**5 (3, 5)**

* Overall score is the sum total of questions 1-7

Supplement 5: Table and figures summarising initial appraisals of all plots by outcome type

Figure A.31: Multiple time-to-event outcomes

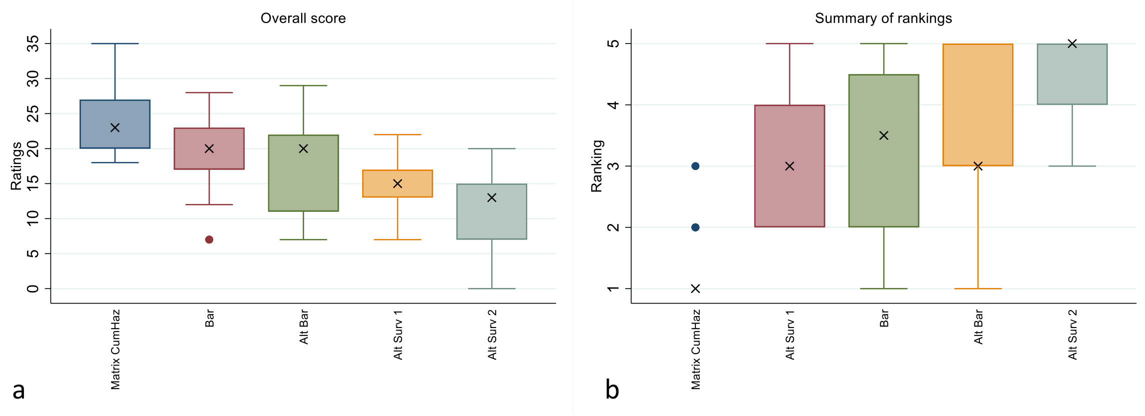

a. Box plot of overall scores ordered by highest to lowest mean values (higher scores indicate better performance). **b. Box plot of rankings** ordered by best to worst mean rank (lower ranking indicates preferred plot).

Note: X indicates median values.

Supplement 5: Table and figures summarising initial appraisals of all plots by outcome type

Table A.5: Plots suitable for **Single TTE Outcomes –** summary of scores

Question	Cumulative Hazard		Kaplan Meier		Mean Cumulative Function		Mean Cumulative Duration		Stacked bar chart over time		Histogram of counts over time	
	n	Mean (SD)	n	Mean (SD)	n	Mean (SD)	n	Mean (SD)	n	Mean (SD)	n	Mean (SD)
		Median (Min, Max)		Median (Min, Max)		Median (Min, Max)		Median (Min, Max)		Median (Min, Max)		Median (Min, Max)
1.Effect size	23	3.1 (1.2)	23	3.2 (1.2)	23	3.0 (1.2)	23	2.6 (1.2)	23	2.2 (1.3)	23	2.0 (1.1)
		3 (1, 5)		4 (1, 5)		3 (1, 5)		3 (1, 4)		2 (1, 5)		2 (1, 5)
2.Direction of effect	23	3.8 (1.1)	23	3.8 (1.1)	23	3.7 (1.1)	23	3.2 (1.2)	23	2.7 (1.3)	23	2.5 (1.2)
		4 (1, 5)		4 (1, 5)		4 (1, 5)		3 (1, 5)		3 (1, 5)		2 (1, 4)
3.Uncertainty	23	4.0 (1.1)	23	4.0 (1.0)	23	3.6 (0.9)	23	3.7 (1.0)	23	1.2 (0.5)	23	1.1 (0.5)
		4 (1, 5)		4 (1, 5)		4 (1, 5)		4 (1, 5)		1 (1, 3)		1 (1, 3)
4.Supplementary data needed	23	3.7 (0.9)	23	3.8 (0.9)	23	2.9 (1.0)	23	2.2 (1.1)	23	2.6 (1.2)	23	2.6 (1.3)
		4 (2, 5)		4 (2, 5)		3 (1, 4)		2 (1, 4)		3 (1, 5)		3 (1, 4)
5.Understandable	23	4.2 (0.7)	23	4.3 (0.7)	23	3.2 (1.1)	23	2.8 (1.1)	23	3.4 (1.2)	23	3.7 (1.1)
		4 (3, 5)		4 (3, 5)		3 (1, 5)		3 (1, 5)		4 (1, 5)		4 (1, 5)
6.Understandable non-stats	23	3.1 (0.9)	23	3.5 (0.8)	23	2.7 (1.1)	23	2.3 (1.0)	23	3.2 (1.1)	23	3.4 (1.2)
		3 (1, 5)		4 (2, 5)		3 (1, 4)		2 (1, 4)		3 (1, 5)		3 (1, 5)
7.Multi-arm studies	21	3.9 (0.5)	21	3.9 (0.5)	21	3.8 (0.9)	21	3.5 (0.9)	21	3.9 (0.7)	21	3.4 (0.9)
		4 (3, 5)		4 (3, 5)		4 (1, 5)		4 (1, 5)		4 (3, 5)		4 (1, 5)
8.Limits numbers	23	3.1 (1.4)	23	3.1 (1.5)	23	3.4 (1.5)	23	3.2 (1.5)	22	2.8 (1.2)	23	3.5 (1.5)
		3 (1, 5)		3 (1, 5)		4 (1, 5)		3 (1, 5)		3 (1, 5)		4 (1, 5)
**9.Overall score**	**21**	**26.1 (3.8)**	**21**	**26.7 (3.6)**	**21**	**23.2 (5.6)**	**21**	**21.0 (5.1)**	**21**	**19.8 (5.6)**	**21**	**19.1 (5.2)**
		**27 (18, 32)**		**27 (18, 32)**		**24 (7, 32)**		**22 (7, 28)**		**18 (11, 32)**		**19 (7, 31)**
10. Suitable for publication	22	3.6 (1.1)	22	4.0 (0.8)	22	3.5 (1.1)	22	3.0 (1.0)	22	2.9 (1.2)	22	2.0 (1.0)
		4 (2, 5)		4 (3, 5)		4 (1, 5)		3 (1, 4)		3 (1, 5)		2 (1, 4)
11. Suitable for final report	22	4.0 (1.0)	22	4.2 (0.7)	22	3.6 (1.1)	22	3.2 (1.1)	22	3.0 (1.2)	22	2.4 (1.0)
		4 (2, 5)		4 (3, 5)		4 (1, 5)		4 (1, 5)		3 (1, 5)		2 (1, 4)
12. Suitable for interim analysis	22	4.0 (1.0)	22	4.2 (0.8)	22	3.3 (1.1)	22	3.0 (1.0)	22	2.8 (1.4)	22	2.9 (1.3)
		4 (2, 5)		4 (2, 5)		3 (1, 5)		3 (1, 5)		3 (1, 5)		3 (1, 5)
13.Exploratory analysis	22	4.0 (0.8)	23	4.0 (1.0)	23	3.6 (0.9)	23	3.5 (0.9)	23	3.2 (1.2)	23	3.3 (1.3)
		4 (2, 5)		4 (1, 5)		4 (1, 5)		4 (1, 5)		3 (1, 5)		4 (1, 5)
14.Explanatory analysis	22	4.0 (1.0)	23	4.2 (0.7)	23	3.5 (1.0)	23	3.0 (1.0)	23	2.7 (1.2)	23	2.3 (1.1)
		4 (2, 5)		4 (3, 5)		4(1, 5)		3 (1, 5)		3 (1, 5)		2 (1, 4)
**Ranking**	**20**	**2.5 (1.5)**	**20**	**1.9 (1.1)**	**19**	**2.9 (1.4)**	**20**	**4.3 (1.6)**	**16**	**4.3 (1.8)**	**18**	**4.8 (1.4)**
		**2 (1, 6)**		**2 (1, 5)**		**3 (1, 5)**		**5 (1, 6)**		**5 (1, 7)**		**5 (2, 7)**

*** Overall score is the sum total of questions 1-7**

Supplement 5: Table and figures summarising initial appraisals of all plots by outcome type

Figure A.32: Single time-to-event outcomes

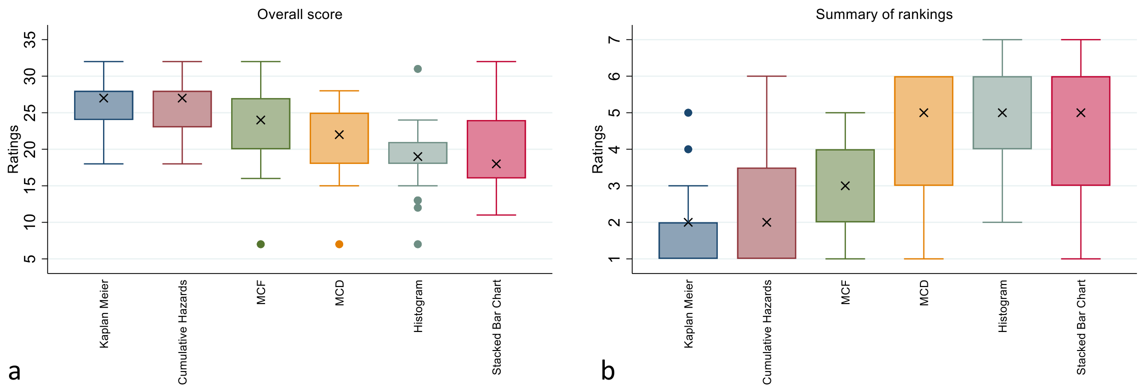

Box plot of overall scores ordered by highest to lowest mean values (higher scores indicate better performance). **b. Box plot of rankings** ordered by best to worst mean rank (lower ranking indicates preferred plot).
Note: X indicates median values.

Supplement 5: Table and figures summarising initial appraisals of all plots by outcome type

Table A.6: Plots suitable for **Multiple Continuous Outcomes** – summary of scores

Question	Scatterplot matrix		E-dish	
	n	Mean (SD)<br>Median (Min, Max)	n	Mean (SD)<br>Median (Min, Max)
1.Effect size	22	2.5 (1.1)	20	1.9 (0.9)
		2 (1, 5)		2 (1, 4)
2.Direction of effect	22	3.0 (1.1)	20	2.5 (1.1)
		3 (1, 5)		2 (1, 5)
3.Uncertainty	22	1.7 (0.8)	20	1.6 (0.8)
		2 (1, 3)		1 (1, 3)
4.Supplementary data needed	22	3.2 (1.1)	20	2.4 (1.1)
		3 (1, 5)		2 (1, 5)
5.Understandable	22	4.5 (0.8)	20	3.8 (1.3)
		5 (2, 5)		4 (1, 5)
6.Understandable non-stats	22	4.3 (0.6)	20	3.6 (1.0)
		4 (3, 5)		4 (1, 5)
7.Multi-arm studies	20	2.8 (1.4)	18	2.2 (1.3)
		3 (1, 5)		2 (1, 5)
8.Limits numbers	22	2.9 (1.2)	19	2.1 (1.2)
		3 (1, 5)		2 (1, 5)
**9.Overall score**	**20**	**22.8 (4.3)**	18	18.6 (5.4)
		**23 (16, 31)**		18 (9, 30)
10. Suitable for publication	20	2.8 (1.3)	19	2.7 (1.2)
		3 (1, 5)		3 (1, 5)
11. Suitable for final report	20	3.4 (1.3)	19	3.2 (1.2)
		4 (1, 5)		3 (1, 5)
12. Suitable for interim analysis	20	4.0 (1.0)	19	3.4 (1.2)
		4 (1, 5)		4 (1, 5)
13.Exploratory analysis	20	4.2 (0.8)	19	3.7 (1.1)
		4 (3, 5)		4 (1, 5)
14.Explanatory analysis	20	3.0 (1.1)	19	2.7 (1.2)
		3 (1, 5)		3 (1, 5)
**Ranking**	**18**	**1.2 (0.4)**	**18**	**1.9 (0.6)**
		**1 (1, 2)**		**2 (1, 3)**

* Overall score is the sum total of questions 1-7

Excludes summary for vector plots as deemed not applicable during the meeting.

Supplement 5: Table and figures summarising initial appraisals of all plots by outcome type

Figure A.33: Multiple continuous outcomes

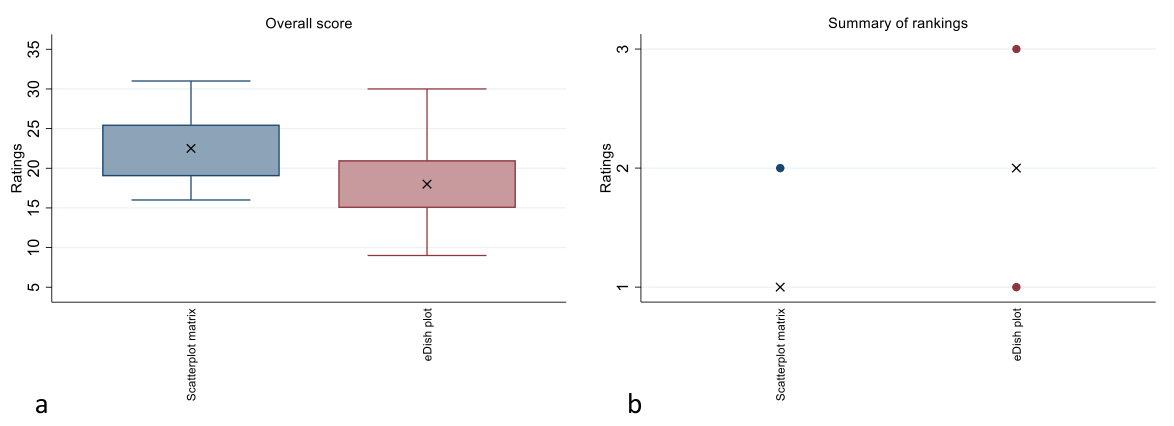

Box plot of overall scores ordered by highest to lowest mean values (higher scores indicate better performance). **b. Box plot of rankings** ordered by best to worst mean rank (lower ranking indicates preferred plot).
Note: X indicates median values. Excludes summary for vector plots.

Supplement 5: Table and figures summarising initial appraisals of all plots by outcome type

Table A.7a: Plots suitable for **Single Continuous Outcomes –** summary of scores

Question	Empirical distribution of max change		Histogram of max change		Delta plot		Line graph - change		Boxplot - change		Violin plot - change	
	n	Mean (SD)<br>Median (Min, Max)	n	Mean (SD)<br>Median (Min, Max)	n	Mean (SD)<br>Median (Min, Max)	n	Mean (SD)<br>Median (Min, Max)	n	Mean (SD)<br>Median (Min, Max)	n	Mean (SD)<br>Median (Min, Max)
1.Effect size	22	2.2 (1.2)	22	2.5 (1.2)	23	1.7 (1.1)	22	3.4 (1.4)	22	2.7 (1.3)	22	2.5 (1.3)
		2 (1, 4)		2 (1, 5)		1 (1, 5)		4 (1, 5)		3 (1, 5)		2 (1, 5)
2.Direction of effect	22	3.1 (1.2)	22	3.0 (1.1)	23	1.6 (0.8)	22	4.0 (0.9)	22	3.1 (1.1)	22	3.0 (1.1)
		3 (1, 5)		3 (1, 4)		1 (1, 4)		4 (2, 5)		3 (1, 5)		3 (1, 5)
3.Uncertainty	22	1.3 (0.5)	22	1.7 (1.0)	23	1.2 (0.4)	22	3.9 (0.9)	22	3.3 (1.3)	22	3.0 (1.1)
		1 (1, 2)		1 (1, 4)		1 (1, 2)		4 (2, 5)		4 (1, 5)		3 (1, 5)
4.Supplementary data needed	22	2.5 (1.1)	22	3.5 (0.9)	23	1.7 (0.8)	22	3.6 (1.0)	22	3.4 (0.9)	22	3.1 (0.8)
		3 (1, 4)		4 (2, 5)		2 (1, 3)		4 (2, 5)		4 (2, 5)		3 (2, 4)
5.Understandable	21	3.0 (0.9)	22	4.5 (0.7)	23	1.7 (0.8)	22	4.4 (0.7)	22	4.5 (0.7)	22	3.8 (0.7)
		3 (1, 4)		5 (3, 5)		2 (1, 4)		4 (3, 5)		5 (3, 5)		4 (3, 5)
6.Understandable non-stats	22	2.4 (0.9)	22	4.2 (0.6)	23	1.4 (0.6)	22	4.1 (0.8)	22	3.9 (0.8)	22	2.9 (0.9)
		3 (1, 4)		4 (3, 5)		1 (1, 3)		4 (3, 5)		4 (2, 5)		3 (2, 5)
7.Multi-arm studies	21	3.9 (0.9)	20	3.2 (0.9)	21	2.4 (1.1)	20	4.0 (0.6)	20	4.0 (0.8)	19	3.7 (0.6)
		4 (1, 5)		3 (2, 5)		2 (1, 4)		4 (3, 5)		4 (2, 5)		4 (3, 5)
8.Limits numbers	22	2.5 (1.6)	20	2.4 (1.5)	22	2.0 (1.2)	22	2.6 (1.4)	22	2.7 (1.4)	22	2.7 (1.4)
		3 (1, 5)		2 (1, 5)		2 (1, 5)		3 (1, 5)		3 (1, 5)		3 (1, 5)
**9.Overall score**	**22**	**17.7 (5.9)**	**21**	**21.9 (5.8)**	**21**	**12.0 (3.8)**	**21**	**26.3 (7.3)**	**21**	**23.7 (6.3)**	**21**	**20.3 (5.4)**
		**19 (0, 26)**		**21 (0, 28)**		**11 (7, 20)**		**28 (0, 35)**		**25 (0, 34)**		**21 (0, 26)**
10. Suitable for publication	22	2.9 (1.2)	21	3.2 (1.0)	21	1.6 (0.6)	21	4.0 (0.7)	21	3.6 (1.0)	21	3.4 (0.9)
		3 (1, 5)		3 (1, 5)		2 (1, 3)		4 (3, 5)		4 (1, 5)		3 (1, 5)
11. Suitable for final report	22	3.1 (1.1)	21	3.7 (1.0)	21	1.8 (0.9)	21	4.0 (0.7)	21	3.6 (1.1)	21	3.4 (1.0)
		4 (1, 5)		4 (1, 5)		2 (1, 4)		4 (3, 5)		4 (1, 5)		3 (1, 5)
12. Suitable for interim analysis	22	3.3 (1.2)	21	3.6 (1.1)	21	2.0 (1.1)	21	3.9 (0.8)	21	3.8 (1.0)	21	3.5 (0.9)
		4 (1, 5)		4 (1, 5)		2 (1, 5)		4 (3, 5)		4 (1, 5)		3 (1, 5)
13.Exploratory analysis	22	3.6 (1.1)	21	3.7 (0.9)	21	2.4 (1.1)	21	4.0 (0.8)	21	3.9 (1.0)	21	3.7 (0.7)
		4 (1, 5)		4 (2, 5)		2 (1, 5)		4 (2, 5)		4 (1, 5)		4 (2, 5)
14.Explanatory analysis	22	2.9 (1.2)	21	3.4 (1.1)	21	1.8 (0.8)	21	4.0 (0.7)	21	3.7 (1.1)	21	3.5 (0.8)
		3 (1, 5)		3 (2, 5)		2 (1, 3)		4 (2, 5)		4 (1, 5)		3 (2, 5)
**Ranking**	**17**	**6.8 (1.9)**	**17**	**4.9 (2.1)**	**19**	**8.1 (1.8)**	**20**	**2.0 (1.2)**	**18**	**3.6 (2.1)**	**18**	**3.8 (1.8)**
		**8 (3, 9)**		**5 (1, 8)**		**9 (2, 9)**		**2 (1, 4)**		**4 (1, 7)**		**4 (1, 7)**

* Overall score is the sum total of questions 1-7

Supplement 5: Table and figures summarising initial appraisals of all plots by outcome type

Table A.7b: Plots suitable for **Single Continuous Outcomes –** summary of scores

**Question**	**Line graph - raw**		**Box plot - raw**		**Violin - raw**	
	n	Mean (SD)<br>Median (Min, Max)	n	Mean (SD)<br>Median (Min, Max)	n	Mean (SD)<br>Median (Min, Max)
1.Effect size	22	3.0 (1.4)	22	2.6 (1.3)	22	2.4 (1.3)
		3 (1, 5)		2 (1, 5)		2 (1, 5)
2.Direction of effect	22	3.7 (1.1)	22	3.0 (1.0)	22	2.9 (1.0)
		4 (1, 5)		3 (1, 5)		3 (1, 4)
3.Uncertainty	22	3.6 (1.0)	22	3.3 (1.3)	22	3.0 (1.1)
		4 (1, 5)		4 (1, 5)		3 (1, 5)
4.Supplementary data needed	22	3.4 (1.1)	22	3.2 (1.0)	22	3.0 (1.0)
		3 (2, 5)		3 (2, 5)		3 (1, 5)
5.Understandable	22	4.5 (0.6)	22	4.4 (0.8)	22	3.8 (0.9)
		5 (3, 5)		5 (2, 5)		4 (2, 5)
6.Understandable non-stats	22	4.2 (0.7)	22	3.9 (0.9)	22	2.9 (1.0)
		4 (3, 5)		4 (2, 5)		3 (1, 5)
7.Multi-arm studies	20	4.0 (0.6)	20	3.9 (0.7)	19	3.6 (0.6)
		4 (3, 5)		4 (2, 5)		4 (3, 5)
8.Limits numbers	22	2.6 (1.4)	22	2.7 (1.4)	22	2.6 (1.4)
		2 (1, 5)		3 (1, 5)		3 (1, 5)
**9.Overall score**	**21**	**25.3 (7.4)**	**21**	**23.1 (6.4)**	**21**	**20.1 (5.4)**
		**25 (0, 35)**		**23 (0, 34)**		**21 (0, 26)**
10. Suitable for publication	21	3.8 (1.0)	21	3.4 (1.1)	21	3.2 (1.0)
		4 (1, 5)		3 (1, 5)		3 (1, 5)
11. Suitable for final report	21	3.9 (1.0)	21	3.6 (1.0)	21	3.4 (1.0)
		4 (1, 5)		4 (1, 5)		3 (1, 5)
12. Suitable for interim analysis	21	3.8 (1.0)	21	3.6 (1.0)	21	3.3 (1.0)
		4 (1, 5)		4 (1, 5)		3 (1, 5)
13.Exploratory analysis	21	3.9 (0.8)	21	3.8 (0.9)	20	3.6 (0.7)
		4 (2, 5)		4 (1, 5)		4 (2, 5)
14.Explanatory analysis	21	3.8 (1.0)	21	3.5 (1.1)	20	3.3 (0.9)
		4 (2, 5)		3 (1, 5)		3 (2, 5)
**Ranking**	**18**	**3.2 (1.8)**	**18**	**4.7 (2.2)**	**18**	**4.7 (2.3)**
		**3 (1, 8)**		**5 (1, 8)**		**5 (1, 8)**

* Overall score is the sum total of questions 1-7

Supplement 5: Table and figures summarising initial appraisals of all plots by outcome type

Figure A.34: Single continuous outcomes

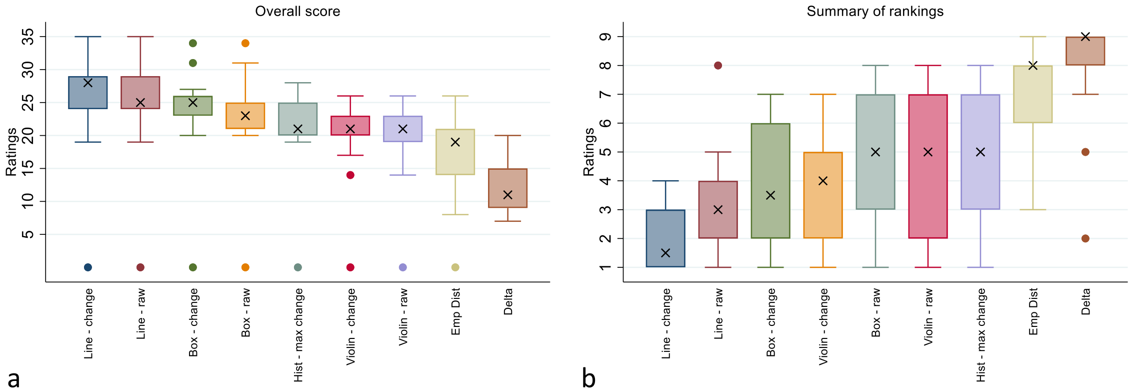

a. Box plot of overall scores ordered by highest to lowest mean values (higher scores indicate better performance). **b. Box plot of rankings ordered** by best to worst mean rank (lower ranking indicates preferred plot).
Note: X indicates median values.
