# Supplementary material for: Visualising harms in publications of randomised controlled trials: consensus and recommendations
Source: BMJ. 2022 May 16;377:e068983. doi: 10.1136/bmj-2021-068983 (PMC9108928; doi:10.1136/bmj-2021-068983)
Supplement: Supplementary file 6 — Web appendix: Supplement 6: Tables summarising Mentimeter votes to decide which plots to take forward and amendments [file phir068983.ww6.pdf]

Supplement 6: Tables summarising Mentimeter votes to decide which plots to take forward and amendments

Table A.8: Decisions for **Multiple Binary Outcomes**

Question		n	%
Should we **keep** the dot plot?	Yes	19	100
	No	0	0
Should we **keep** the stacked bar chart?	Yes	18	95
	No	1	5
Should we **keep** the bar chart?	Yes	17	81
	No	4	19
Should we **exclude** the tendril plot?	Yes	20	100
	No	0	0
Should we **exclude** the alluvial plot?	Yes	17	94
	No	1	6
Should we **exclude** alternative 3?	Yes	18	100
	No	0	0
Should we **keep** the volcano plot?	Yes	11	65
	No	6	35
Should we **keep** alternative 1?	Yes	8	47
	No	9	53
Should we **keep** alternative 2?	Yes	10	50
	No	10	50
Should we keep the **heat** map?	Yes	3	16
	No	16	84
Should we keep the **star** plot?	Yes	2	10
	No	18	90

Supplement 6: Tables summarising Mentimeter votes to decide which plots to take forward and amendments

Table A.9: Decisions about amendments for the recommend plots for **Multiple Binary Outcomes**

		n	%
Are we happy to recommend the dot plot as it is unedited?	Yes	14	67
	No	7	33
Do we want to add in counts and number of participants into the data table?	Yes	12	60
	No	8	40
Are we happy to recommend the stacked bar chart as it is unedited?	Yes	16	80
	No	4	20
Do we want to recommend the volcano in light of possible alternative?	Yes	7	35
	No	13	65
	Yes	7	35
Do we want to recommend the alternative 2 instead?	No	13	65

Table A.10: Decisions about plots in the **Single Binary Outcome** setting

		n	%
Is it helpful to use a plot in this setting?	Yes	14	67
	No	7	33
Would you like to see this in bar chart?	Yes	15	75
	No	5	25
Would you prefer the data to be presented by bars or dots?	Bars	15	79
	Dots	4	21
Should we present as two separate charts one above the other aligned vertically?	Yes	9	50
	No	9	50
Should we present as two separate charts one above the other aligned vertically?	Context specific e.g. only 2 arms then horizontal, >2 arms then stacked	11	58
	Stacked one above the other	7	37
	Horizontal - side by side	1	5

Supplement 6: Tables summarising Mentimeter votes to decide which plots to take forward and amendments

Table A.11: Decisions for plots to recommend in the **Single Time-to-Event** setting

		n	%
Should we recommend KM or Cumulative Hazard plots?	Cumulative hazard	3	17
	Kaplan-Meier	15	83
What should the table at the bottom of the KM plot contain?	No table	1	6
	Minimum - at risk table only (by arm)	4	24
	Full table as per KMUNICATE	12	71
Should we recommend the survival ratio plot as an alternative to the KM?	Yes	12	67
	No	6	33
Should we recommend the MCF plot for displaying information on repeated events?	Yes	15	88
	No	2	12
Should the table at the bottom of the MCF plot only contain the number at risk (by arm)	Yes	17	94
	No	1	6

Acronyms: KM – Kaplan-Meier; MCF – Mean Cumulative Function

Table A.12: Decisions for plots in the **Multiple Time-to-Event** setting

		n	%
Should we recommend any of these plots?	Matrix of multiple KM	8	40
	Bar chart of median time-to-event	0	0
	Heat map/alternative 4	2	10
	None of these	10	50

Acronyms: KM – Kaplan-Meier

Supplement 6: Tables summarising Mentimeter votes to decide which plots to take forward and amendments

Table A.13: Decisions for plots in the **Single Continuous Outcome** setting

		n	%
Should we recommend a version of the line chart?	Yes	17	94
	No	1	6
Should we recommend a version of the boxplot?	Yes	9	53
	No	8	47
Should we recommend a version of the violin plot?	Yes	12	67
	No	6	33
Should we recommend a version of the histogram?	Kernel density	11	61
	Histogram	0	0
	Neither	7	39

Table A.14: Decisions for plots in the **Multiple Continuous Outcome** setting

		n	%
Should we recommend the scatterplot matrix?	Yes	16	94
	No	1	6
