# Supplementary material for: Visualising harms in publications of randomised controlled trials: consensus and recommendations
Source: BMJ. 2022 May 16;377:e068983. doi: 10.1136/bmj-2021-068983 (PMC9108928; doi:10.1136/bmj-2021-068983)
Supplement: Supplementary file 7 — Web appendix: Supplement 7: Free text comments accompanying initial appraisals of recommended plots [file phir068983.ww7.pdf]

Supplement 7: Free text comments accompanying initial appraisals of recommended plots

1. *Multiple binary outcomes*

    i. *Dot plot*

Proposals to the dot plot included adding numerical raw data via either a data table on the right-hand side of the plot or labelling data points on the left-hand side of the plot in order to enrich information presented and to provide an alternative to the typical frequency tables presented in publications. Concerns were raised about the inclusion of confidence intervals in this plot as this could encourage use as a proxy for hypothesis tests but discussions indicated that this could be caveated by including a caution to avoid such interpretation in the recommendations for use.

    ii. *Stacked bar charts*

Preference was for stacked bar charts of percentages with at least one event and inclusion of bar labels of frequencies or counts of events. Imposing a meaning to the order of bars was also advocated.

2. *Single binary outcomes*

    i. *Bar chart of counts*

A boxplot or dot plot of a summary measure of count data was suggested to replace the bar chart as a means to summarise count data, however, there was not whole group support for this idea. There was variation in preferences for layout of the bar chart with some preferring side-by-side plots for each treatment group and others preferring plots stacked one above the other for each treatment group. Discussions highlighted that some participants question the need for this plot, for example, with one participant commenting, "*is aggregation of data like this helpful?*", and others felt there could be difficulty in interpreting these plots.

Supplement 7: Free text comments accompanying initial appraisals of recommended plots

Discussions concluded that this plot might only be useful for summaries of serious events or pre-specified events.

3. *Single time-to-event outcomes*

   i. *Kaplan-Meier*

Amendments discussed for the Kaplan-Meier plot included: incorporating an extended at risk table including the number of participants that remain 'at risk', the cumulative number that have been censored and the cumulative number that have experienced an event at each discrete time point; providing a clear definition of what 'survival' means in the context of analysing harm outcomes in the recommendations; and incorporating a between group comparison. This latter point prompted discussions and a suggestion to consider survival ratio plots proposed by Newell et al.[245] Survival ratio plots were not formally considered via appraisals but were incorporated into discussions for consideration. Given the context of use we instead refer to the survival ratio plot as the event-free ratio plots. Discussions revealed some concerns about use of time-to-event plots in this setting and concluded that recommendations should caution users to bear in mind the consequences of competing risks.

   ii. *Mean cumulative function*

Proposals for the mean cumulative function included adding confidence interval bands and at risk tables. Discussions covered whether grouping all events together in this plot should be encouraged or that instead the recommendation should be for use when analysing pre-specified events of interest. Participants endorsed the latter, recommending use to account for recurrent events. Discussions also indicated that recommendation should include clear text descriptions explaining the interpretation of this plot given its novelty and clarifying that it adequately accounts for censoring.

Supplement 7: Free text comments accompanying initial appraisals of recommended plots

4. *Multiple time-to-event outcomes*

Discussions on the plots for displaying multiple time-to-event outcomes highlighted the lack of a suitable plot for consideration and that further development work in this area is needed. However, in the interim, initial discussions indicated that the matrix of Kaplan-Meier plots could be utilised and was taken forward for further critique, although ultimately was not recommended.

   i. *Matrix of Kaplan-Meier*

Discussions indicated that the matrix of Kaplan-Meier plots required incorporation of confidence bands and tables of numbers at risk as per the individual Kaplan-Meier plots. Participants indicated that this plot would be useful to detect disproportionalities for pre-specified events but that the number of events looked at would need to be limited to be useful. To avoid encouraging performance of many hypothesis tests it was also highlighted that it should be clearly specified that this plot should be used as a way to display risk over time to help identify disproportionalities and raise signals for adverse drug reactions. Alternatives that incorporate information on recurrent events are still needed.

5. *Multiple continuous outcomes*

   i. *Scatterplot matrix*

Discussed amendments to the scatterplot matrix included ways to help ease the problems created by overlapping points, inclusion of reference lines and labels for outliers.

Supplement 7: Free text comments accompanying initial appraisals of recommended plots

6. *Single continuous outcomes*

   i. *Line chart*

Discussions focused on the appropriate statistic to display on the line chart as well as advocating for inclusion of tables with numbers at risk at the bottom of the plot that are typically seen on time-to-event plots such as the Kaplan-Meier plot.

   ii. *Violin plot*

A proposal to remove the duplication of information in the ‘mirrored’ distributions of the violin plot was discussed and an indication of a preference for violin plots over box plots was voiced.

   iii. *Histogram/Kernel density plot*

Discussions indicated that participants wished to see this information presented graphically but would prefer to see it displayed in kernel density plots instead of histograms, which would overcome the problems of overlap encountered in the histogram.
